# Full Manure Recycling Risks an 18% Rise in China's Cropland N_2_O Emissions Without Improved Management

**DOI:** 10.1111/gcb.70837

**Published:** 2026-04-12

**Authors:** Shidi Ba, Zhaohai Bai, Lin Ma, Davey L. Jones, David R. Chadwick

**Affiliations:** ^1^ School of Environmental and Natural Sciences Bangor University Bangor UK; ^2^ Key Laboratory of Agricultural Water Resources, Center for Agricultural Resources Research Institute of Genetics and Developmental Biology, Chinese Academy of Sciences Shijiazhuang China; ^3^ State Key Laboratory of Pollution Control and Resource Reuse, School of the Environment Nanjing University Nanjing China

**Keywords:** boosted regression tree model, Chinese cropland, fertilization, machine learning, manure substitution, N_2_O‐N loss

## Abstract

Cropland fertilization is the largest anthropogenic source of nitrous oxide (N_2_O‐N) emissions in China and could be mitigated through manure substitution. However, accurate quantification of N_2_O‐N losses under different combinations of manure and chemical fertilizer applications remains challenging due to limited national data, oversimplified methods of estimation, and neglect of the nitrogen (N) source (chemical‐N and manure‐N) interaction effects under mixed fertilization on N_2_O‐N production processes. Using a dataset consisting of 2186 observations across China, we developed N_2_O‐N models for fertilized (chemical fertilizer and manures) croplands incorporating climate, soil, fertilization and cropping variables, with particular emphasis on the manure component, through machine‐learning approaches. Model simulations estimated a median N_2_O‐N flux of 1.1 kg ha^−1^ and a total national N_2_O‐N loss of 243.7 Gg in 2019, with a hotspot in Northcentral China. During 2000–2019, N_2_O‐N losses were driven mainly by annual total N input. By 2050, merely pursuing 100% manure recycling without considering improved manure‐N use efficiency could lead to an 18% increase in N_2_O‐N losses compared with 2019. Hence, this study provides a high‐resolution modeling framework to predict how developments in manure substitution will affect the magnitude and spatial distribution of N_2_O‐N losses. We also demonstrate that future manure substitution policies will need to promote improved manure nutrient management at the same time as greater manure recycling rates.

## Graphical abstract

By 2050, merely pursuing 100% manure recycling without improving manure‐N use efficiency could lead to an 18% increase in N_2_O‐N losses compared with 2019. This challenges China's current policy focus solely on manure recycling quantity, as reflected in the “14th Five‐Year Plan for National Agricultural Green Development” (2021–2025). By highlighting the need to promote greater manure recycling rate alongside improved manure nutrient management, this study provides timely guidance for sustainable policy refinement.

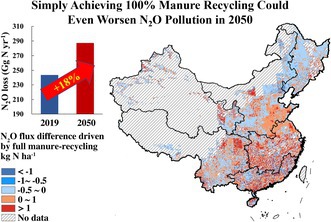

## 1 Introduction

Nitrous oxide (N_2_O) represents a potent greenhouse gas contributing significantly to global warming and stratospheric ozone depletion, with a unit warming potential of 265–298 CO_2_‐equivalent over a 100‐year time horizon (IPCC 2021). Increasing evidence indicates that China is currently the largest emitter of anthropogenic nitrogen (N) through N_2_O, with annual emissions reaching 1107 Gg N_2_O‐N yr.^−1^ (Liang et al. 2024; Zhou et al. 2014), contributing 15.2% of the global total (Liang et al. 2024). Among this, 31.8% (27.5% and 4.3% from chemical fertilizers and manure application, respectively) is attributed to national cropland fertilization (Liang et al. 2024), primarily due to the overuse of chemical fertilizers, improper use of manures, and low nitrogen use efficiency (NUE) in cropping systems (He et al. 2018; Huang et al. 2025; Liu et al. 2024). Numerous studies have confirmed that substituting chemical fertilizers with manure is a key component of sustainable agricultural practices, offering the potential to reduce chemical fertilizer production and use, improve crop N uptake and soil health, and minimize nutrient losses to the environment (Du et al. 2020; Ren et al. 2022; Zhang et al. 2020; Zhou et al. 2016). However, the agronomic value of manure remains undervalued in China (Chadwick et al. 2015; Sun et al. 2020). In 2022, although China's extensive livestock industry produced nearly 13.3 Tg of manure‐N, almost half the national chemical‐N usage (24.6 Tg), inefficiencies and losses during housing, storage, processing, and discharge stages meant that only about 4.9 Tg was ultimately recycled to croplands as effective manure‐N input (Bai et al. 2016; FAOSTAT 2024; Niu and Ju 2017; Zhang et al. 2020). Thus, it is essential to accurately quantify N_2_O‐N loss from Chinese cropland under different combinations of manure and chemical fertilizer applications to assess the potential benefits of substituting manure for chemical fertilizer N and to better assess China's progress toward sustainable agriculture.

China has a vast and diverse agricultural landscape characterized by contrasting climate conditions, soil properties, and cropping systems (Yue et al. 2019; Zhou, Shang, et al. 2015). Estimating N_2_O‐N losses under mixed fertilization (e.g., the combined use of chemical fertilizer and manure) is notoriously challenging due to the considerable complexity and high spatiotemporal variability associated with the multiple interacting factors (Paustian et al. 2016; Tian, Yang, et al. 2018). Over the past decades, several empirical emission factor (EF) models (e.g., PKU‐N_2_O, NUFER, IAP‐N, STIRPAT and CHEN), process‐based models (e.g., DNDC, APSIM, SPACSYS, TRIPLEX‐GHG, DLEM, VISIT and WHCNS‐Veg) and statistical models (e.g., SRMN and N_2_O_STAT) have been employed to estimate cropland N_2_O‐N losses under mixed fertilization scenarios in China. However, the substantial variation in validation performance (*R* ^2^ values ranging from 0.20 to 0.92) demonstrates that robust prediction of N_2_O‐N emissions under mixed fertilization remains challenging. This inconsistency stems primarily from three issues: (i) First, many existing models (including those process‐based models and N_2_O_STAT) were primarily developed and parameterized using limited regional data or data from studies outside of China, potentially compromising their accuracy in reflecting nationwide N_2_O‐N emissions (Wang, Zhou, et al. 2020). (ii) Second, empirical and process‐based approaches often rely aggressively on fixed EFs, coefficients, or equations to represent fertilization responses, which may oversimplify non‐linearities and context‐dependent effects, thereby increasing uncertainty in N_2_O‐N estimates from China's heterogeneous croplands (Albanito et al. 2017; Wang, Zhou, et al. 2020). (iii) In addition, numerous studies have reported that equivalent amounts of pure‐N (chemical‐N or manure‐N) versus mixed‐N inputs (including chemical‐N and manure‐N) produce markedly different N_2_O‐N emissions (Ren et al. 2017; Zhang et al. 2020), which are significantly influenced by the ratio of chemical‐N to manure‐N inputs (Xia et al. 2017). This suggests that mixed fertilization requires distinct treatment in models compared with chemical‐N or manure‐N only scenarios. However, current modeling frameworks (e.g., those empirical‐EF models and N_2_O_STAT) often only estimate N_2_O‐N emissions under mixed fertilization by treating the two N sources separately and then summing the emissions from each component, implicitly assuming additivity (Aneja et al. 2019; Bu et al. 2024; Xu et al. 2020). This simplification may fail to capture source‐interaction effects under mixed fertilization, such as mineralization timing, carbon availability, soil property and microbial activity (Xu et al. 2021; Zhang et al. 2020), and thereby may hinder quantification of actual cropland N_2_O‐N loss patterns. Hence, there is an urgent need to develop up‐to‐date, region‐specific datasets and implement enhanced modeling approaches that enable comprehensive attribution and precise assessment of N_2_O‐N fluxes from croplands fertilized with chemical fertilizer and manure throughout China, particularly under mixed fertilization applications.

Recently developed machine learning algorithms utilizing data‐driven methodologies have emerged as powerful tools for estimating soil biogeochemical processes (Pan et al. 2021), demonstrating superior performance compared to traditional approaches in predicting soil N_2_O‐N losses (Saha et al. 2021; Villa‐Vialaneix et al. 2012). Machine learning techniques can improve integration of heterogeneous input datasets and capture nonlinear relationships between N transformations and multiple interacting environmental factors, thereby providing a robust and innovative framework for accurately predicting national‐scale N_2_O‐N losses from N inputs to croplands (Breiman 1996; Friedman 2001, 2002).

Through the integration of a comprehensive data compilation with spatially explicit grid‐level datasets, this study represents the first application of multiple machine learning models to accurately evaluate N_2_O‐N emissions from N inputs via chemical fertilizer and manures across China's croplands, particularly under mixed fertilization conditions. The objectives of this study were to (1) characterize the spatial distribution and temporal dynamics of cropland N_2_O‐N losses following N input in China from 2000 to 2019; (2) determine the relative contributions of key driving factors to national trends in N_2_O‐N emissions from cropland during this timeframe; and (3) assess the potential benefits of nationwide implementation of optimized manure substitution for future N_2_O emission mitigation.

## 2 Materials and Methods

**FIGURE 1:**
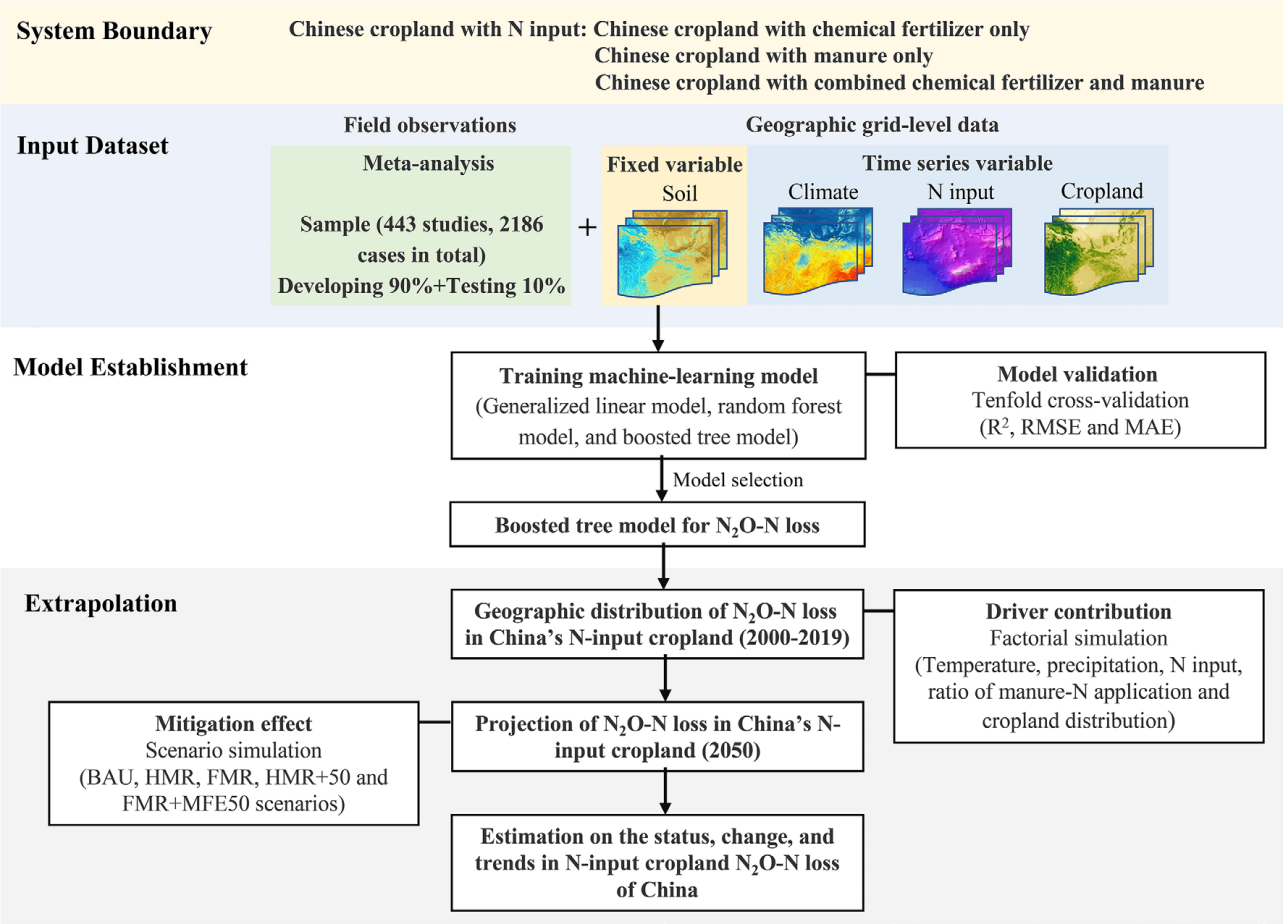
Flowchart for quantifying cropland N_2_O‐N losses in China. Regression coefficients of determination (*R* ^2^); root mean square error (RMSE); mean absolute error (MAE); baseline business as usual scenario (BAU); half manure recycling scenario (HMR); full manure recycling scenario (FMR); the scenario combining half manure recycling with manure‐N fertilizer equivalency improved to 50% (HMR + MFE50) and the scenario combining full manure recycling with manure‐N fertilizer equivalency improved to 50% (FMR + MFE50).

This study included a variety of approaches. Modeling procedures were programmed in R Studio v. 4.2.0, and the following maps were plotted by ArcGIS v. 10.8.1 software. The flowchart of data processing and analysis is shown in Figure 1.

Our estimates specifically focused on fertilizer‐induced N_2_O‐N emissions to emphasize the direct impact of manure substitution strategies, and therefore did not include contributions from other N sources, such as human excreta (Luo et al. 2019), crop residues (Gao et al. 2011; Luo et al. 2019), irrigation‐N (He et al. 2018), and atmospheric‐N deposition (He et al. 2018).

### 2.1 N_2_O‐N Flux Data

**FIGURE 2:**
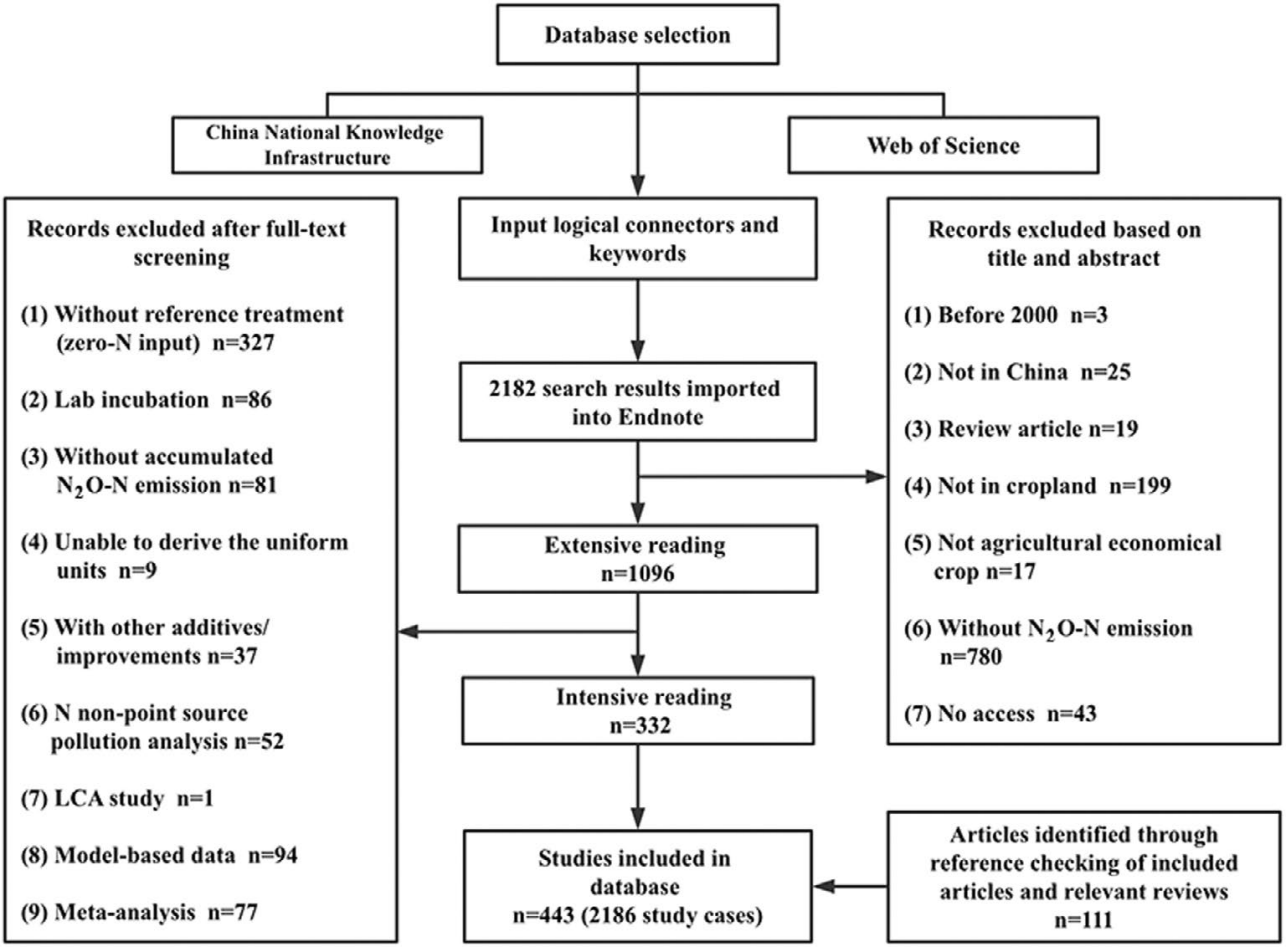
The scientific selection of peer‐reviewed publications.

Peer‐reviewed studies on N_2_O‐N losses from fertilization in Chinese croplands were retrieved from the China National Knowledge Infrastructure (CNKI, http://www.cnki.net) and Web of Science (WOS, http://apps.webofknowledge.com/) databases, covering the period from January 2000 to April 2025. “Fertilization”, “N_2_O‐N loss” and “Chinese cropland” were used as the keywords for the literature search. To ensure the reliability of the collected publications, a systematic selection process was implemented based on a set of inclusion and exclusion criteria (Figure 2). The studies included in this analysis were required to meet the following criteria: (i) the study focused either exclusively on chemical fertilization, or on partial or full substitution of chemical fertilizers with manure; (ii) the experiment was conducted under field conditions in China; (iii) the study reported cumulative N_2_O‐N fluxes for both a zero‐N input treatment (reference treatment) and N input treatments. Based on such criteria, a database comprising 443 published papers with 2186 individual measurements (689 with manure application) across China was compiled. From each selected publication, data were extracted on mean cumulative N_2_O‐N fluxes (kg N ha^−1^) for both reference and N‐added treatments, along with information on potential factors influencing soil N_2_O‐N emission, including: climate—annual mean air temperature (MAT) and annual accumulated precipitation (AAP); background soil properties—total N (TN), total phosphorus (TP), available phosphorus (AP; measured as Olsen‐extractable phosphorus), pH value (pH), soil organic matter (SOM), bulk density (BD); N management—chemical‐N input (CheN), manure‐N input (ManN) and ratio of manure‐N to total N application (ratio of manure‐N application); and cropping system—paddy versus upland (Crop). Graphical data were digitized and extracted using WebPlotDigitizer (Burda et al. 2017).

### 2.2 Model Development and Validation

Given the vast geographic extent of China and the non‐zonal spatial distribution of cropland areas and N inputs, N_2_O‐N flux across all croplands were estimated using machine learning approaches. Accordingly, three distinct machine learning models, e.g., generalized linear model, random forest model, and boosted regression tree, were employed to predict net cumulative N_2_O‐N fluxes (kg N_2_O‐N ha^−1^) under N input conditions. These models were chosen because they represent widely used and well‐established algorithms in the machine learning domain (Lam et al. 2024). A brief description of each model is provided in supporting information (Text S1). These estimations were based on 12 key driving variables, categorized into: (i) climatic factors (MAT and AAP); (ii) soil characteristics (TN, TP, AP, pH, SOM, and BD); (iii) N management practices (CheN, ManN and ratio of manure‐N application); and (iv) cropping system (Crop). The entire N_2_O‐dataset was randomly divided into a 90% development set and a 10% independent test set. Hyperparameters were tuned on the development set using tenfold cross‐validation (ninefold for training and onefold for validation) (Tian et al. 2023). The fitted model was evaluated on the independent test set. Three metrics, including regression coefficients of determination (*R* ^2^), root mean square error (RMSE) and mean absolute error (MAE) (Li et al. 2020; Ren et al. 2023), were used to assess prediction accuracy and identify the best‐performing model (highest *R* ^2^ and the lowest RMSE and MAE). The LOESS‐smoothed partial dependence plots (PDPs) were employed to visualize the relationships between key variables and net cumulative N_2_O‐N flux (Wang et al. 2023). PDPs quantify the effect of an individual variable on N_2_O‐N flux while holding all other variables constant (Greenwell 2018).

### 2.3 Spatial Extrapolation of N_2_O‐N Loss

The selected machine learning model was used to reconstruct the spatial distribution of N_2_O‐N flux in N‐input (via chemical‐N and manure‐N) croplands between 2000 and 2019. The year 2000 was selected as the start year because China began gradually implementing manure‐related policies and regulations from 2000 (Wei et al. 2021). The year 2019 was chosen as the end year because the Harmonized Anthropogenic N Inputs (HaNi) dataset used to develop the spatiotemporal N_2_O‐N maps in this study currently extends only up to 2019 and remains the most up‐to‐date gridded N application dataset available for China with an explicit distinction between chemical‐N and manure‐N input rates (Tian et al. 2022). To explore regional differences, Chinese cropland was classified into six major regions: Northeast (Heilongjiang, Jilin and Liaoning), Northcentral (Beijing, Tianjin, Hebei, Henan, Shandong, Shanxi, Shaanxi and Inner Mongolia), Northwest (Gansu, Ningxia, Qinghai and Xinjiang), Middle and lower of Yangtze river (Jiangsu, Shanghai, Anhui, Zhejiang, Hunan, Hubei and Jiangxi), Southeast (Guangdong, Guangxi, Fujian and Hainan), and Southwest (Sichuan, Chongqing, Yunnan, Guizhou and Tibet). This study did not include Hong Kong, Macao, and Taiwan. The six delineated regions represent major agro‐climatic zones in China and capture the regional heterogeneity in climatic and soil properties (Table S2).

To align with the requirements of the selected model, climate variables (e.g., MAT and AAP) were sourced from the National Tibetan Plateau/Third Pole Environment Data Center (TPDC, http://data.tpdc.ac.cn) (Peng et al. 2019). Soil property data were obtained from the Harmonized World Soil Database (HWSD v2.0, 2023) at a spatial resolution of 1 km. Annual grid‐level application rates of chemical fertilizers and manure in croplands (5‐arcminute resolution) were retrieved from the HaNi dataset (Tian et al. 2022). The spatial distribution of cropland area (Yu et al. 2022) and cropland type (Xu et al. 2018) were acquired from the National Ecosystem Science Data Center (NESDC, https://www.nesdc.org.cn).

### 2.4 Drivers of N_2_O‐N Loss

A series of factorial simulations were performed using the selected machine learning model to quantify the individual contributions of climate and agricultural management changes to the observed trend in N_2_O‐N loss from croplands receiving chemical fertilizer and manure across China. In the baseline simulation (S0), all input variables were allowed to vary from the year 2000 to the target year (Table 1). In each subsequent simulation, one additional driving factor was held constant at its 2000 level, while the remaining drivers continued to vary over time (Table 1). Consequently, the differences between successive simulations, for example, S0 versus S1, S1 versus S2, S2 versus S3, S3 versus S4, and S4 versus S5, isolated the respective effects of MAT, AAP, annual total N input (the sum of CheN and ManN), the annual ratio of manure‐N application, and annual cropland distribution on the trend of N_2_O‐N loss.

**TABLE 1:** Simulation protocol for quantifying the contribution of different drivers to historical changes in N_2_O‐N losses across Chinese cropland.

Driver	Factorial simulation					Contribution
	S0	S1	S2	S3	S4	
MAT	T1–T2	T1	T1	T1	T1	ΔN_2_O‐N (S0–S1)
AAP	T1–T2	T1–T2	T1	T1	T1	ΔN_2_O‐N (S1–S2)
Annual total N input	T1–T2	T1–T2	T1–T2	T1	T1	ΔN_2_O‐N (S2–S3)
Annual ratio of manure‐N application	T1–T2	T1–T2	T1–T2	T1–T2	T1	ΔN_2_O‐N (S3–S4)
Annual cropland distribution	T1–T2	T1–T2	T1–T2	T1–T2	T1–T2	ΔN_2_O‐N (S4–S5)

*Note:* T1 indicates that the driver was held at the same level as in T1 (the initial year of historical records), while T1–T2 indicates that the driver varied from T1 to T2 (the final year of historical records). Annual mean air temperature (MAT); annual accumulated precipitation (AAP); annual total N (the sum of chemical‐N and manure‐N) input (annual total N input); and the annual ratio of manure‐N to total N (the sum of chemical‐N and manure‐N) application (annual ratio of manure‐N application).

### 2.5 Projection of Future N_2_O‐N Loss

By 2050, global animal production is projected to double, growing at a faster rate than any other agricultural subsector, particularly in developing countries (FAOSTAT 2024). This rapid growth will significantly intensify the environmental burden of livestock manure, making mitigation strategies increasingly crucial (FAOSTAT 2024). To assess the future potential for N_2_O‐N mitigation in China through manure substitution, five fertilization scenarios for 2050 were simulated using our trained machine learning model. In each scenario, manure recycling rates and manure‐N fertilizer equivalency (MFE) coefficients (Text S3) jointly defined the manure substitution level required to maintain the same plant‐available N supply (Text S4), where MFE represents manure‐N availability influenced by improvements in manure management (e.g., storage, processing, and spreading) prior to land application (Text S3) (Jensen 2013; Webb et al. 2013). *Business‐as‐usual baseline (BAU) scenario*: To satisfy the increasing food demand projected under the Shared Socioeconomic Pathway 2 (SSP2) by 2050, the amounts of chemical fertilizer and manure inputs were adjusted in accordance with anticipated changes in crop and animal production (Bai et al. 2022; Chen et al. 2011; Jin et al. 2020). However, the manure recycling rate was maintained at its 2019 level, and the MFE value was fixed at 30% in 2050 (Jin et al. 2020), reflecting no additional advancements in manure management. Climate projections were aligned with the SSP2‐4.5 pathway, representing a medium‐level carbon emissions trajectory by 2050. Based on the latest binding targets outlined in the “14th Five Year Plan for National Agricultural Green Development” (2021–2025), which sets the manure recycling rates at 80% by 2025 and 85% by 2035 (Lin et al. 2025), four manure substitution scenarios were developed as follows: *HMR (half manure recycling) and FMR (full manure recycling) scenarios*: Building upon the BAU scenario, HMR and FMR focused on increasing the proportion of manure that is properly collected and recycled back into cropland. These scenarios maintained the same national level of available‐N input and MFE value as the BAU scenario, while ensuring the half (HMR) or full (FMR) application of manure to cropland in 2050. *HMR + MFE50 (HMR with 50% MFE) and FMR + MFE50 (FMR with 50% MFE) scenarios*: These scenarios further optimized HMR and FMR by increasing MFE from 30% to 50% (Jin et al. 2020), representing a conservative and achievable improvement based on the average enhanced‐MFE value summarized from European field studies (Jensen 2013; Ma et al. 2024; Webb et al. 2013), due to the current lack of comparable data from China. This improvement reflects the implementation of advanced manure management techniques to preserve plant‐available N content of manure, such as slurry storage covering, slurry acidification, enclosed composting system and shallow injection, while eliminating the direct discharge of manure into the environment. Further methodological details are presented in Table 2 and Text S4.

**TABLE 2:** Key parameters employed in the historical and 2050 scenario analyses.

	2000	2014	2019	2050 BAU	2050 HMR	2050 FMR	2050 HMR+ MFE50	2050 FMR+ MFE50
*Parameters*								
National manure‐N input								
Manure‐N in excreta (*T*_*g*_)	13.56	12.75	12.43	23.97	23.97	23.97	23.97	23.97
Manure recycling rate (%)	33.73	37.33	35.17	35.17	50.00	100.00	50.00	100.00
Total manure‐N input (*T*_*g*_)	4.57	4.76	4.37	8.43	11.99	23.97	11.99	23.97
Manure‐N fertilizer equivalence value (%)	30.00	30.00	30.00	30.00	30.00	30.00	50.00	50.00
Available manure‐N input (*T*_*g*_)	1.37	1.43	1.31	2.53	3.60	7.19	5.99	11.99
National chemical‐N input								
Available chemical‐N input (*T*_*g*_)	22.14	30.98	23.02	26.06	25.00	21.40	22.60	16.61
National total available‐N input^a^ (*T*_*g*_)	23.51	32.41	24.33	28.59	28.59	28.59	28.59	28.59
National manure‐N substitution ratio (%)	5.84	4.40	5.39	8.85	12.58	25.15	20.96	41.92
*Gridded parameters*								
Chemical fertilization	HaNi for 2000	HaNi for 2014	HaNi for 2019	1.13*(HaNi for 2019)	1.09*(HaNi for 2019)	0.93*(HaNi for 2019)	0.98*(HaNi for 2019)	0.72*(HaNi for 2019)
Manure fertilization	HaNi for 2000	HaNi for 2014	HaNi for 2019	1.93*(HaNi for 2019)	2.74*(HaNi for 2019)	5.49*(HaNi for 2019)	2.74*(HaNi for 2019)	5.49*(HaNi for 2019)
Cropland distribution	NESDC for 2000	NESDC for 2014	NESDC for 2019	LUH2 for 2050 under SSP2‐4.5	LUH2 for 2050 under SSP2‐4.5	LUH2 for 2050 under SSP2‐4.5	LUH2 for 2050 under SSP2‐4.5	LUH2 for 2050 under SSP2‐4.5
Climate condition	TPDC for 2000	TPDC for 2014	TPDC for 2019	WorldClim for 2050 under SSP2‐4.5	WorldClim for 2050 under SSP2‐4.5	WorldClim for 2050 under SSP2‐4.5	WorldClim for 2050 under SSP2‐4.5	WorldClim for 2050 under SSP2‐4.5
Soil property	HWSD for 1980s	HWSD for 1980s	HWSD for 1980s	HWSD for 1980s	HWSD for 1980s	HWSD for 1980s	HWSD for 1980s	HWSD for 1995

*Note:* Baseline business as usual scenario (BAU); half manure recycling scenario (HMR); full manure recycling scenario (FMR); the scenario combining half manure recycling with manure‐N fertilizer equivalency improved to 50% (HMR + MFE50); the scenario combining full manure recycling with manure‐N fertilizer equivalency improved to 50% (FMR + MFE50); harmonized anthropogenic N inputs database (HaNi); national ecosystem science data center (NESDC); land‐use harmonization^2^ database (LUH2); the intermediate‐carbon emissions pathway (SSP2‐4.5); national Tibetan plateau/third pole environment data center (TPDC); WorldClim database (WorldClim); harmonized world soil database (HWSD). Parameters were derived from Xu et al. (2018), Peng et al. (2019), Tian et al. (2022), Yu et al. (2022), Bai et al. (2022), Jin et al. (2020), and FAOSTAT (2024).

^a^ This is the sum of the available manure N and available chemical fertilizer. The asterisk (*) presented in Table 2 denotes multiplication, not statistical significance.

To support future scenario simulations, datasets for 2050 climate, N input, and cropland area were needed. High‐resolution climate data (30 arc‐seconds) for 2050 were retrieved from the SSP2‐4.5 scenario using the EC‐Earth3‐Veg climate model, as provided in the WorldClim 2.1 (http://www.worldclim.org). Furthermore, grid‐level chemical‐N and manure‐N application rates for 2050 were derived by proportionally scaling the 2019 baseline maps from the Anthropogenic Nitrogen Inputs (HaNi) database (Tian et al. 2022), based on coupled projections from the GLOBIOM‐China model and NUFER model (Text S2) following the SSP2 pathway (Bai et al. 2022; Chen et al. 2011; Jin et al. 2020; Zhao et al. 2021). Cropland area at the grid level for 2050 was sourced from the SSP2‐4.5 storyline within the Land‐Use Harmonization2 dataset (LUH2 v2f, https://luh.umd.edu/index.shtml).

## 3 Results

### 3.1 Influencing Factors and Model Performance of N_2_O‐N Flux

**FIGURE 3:**
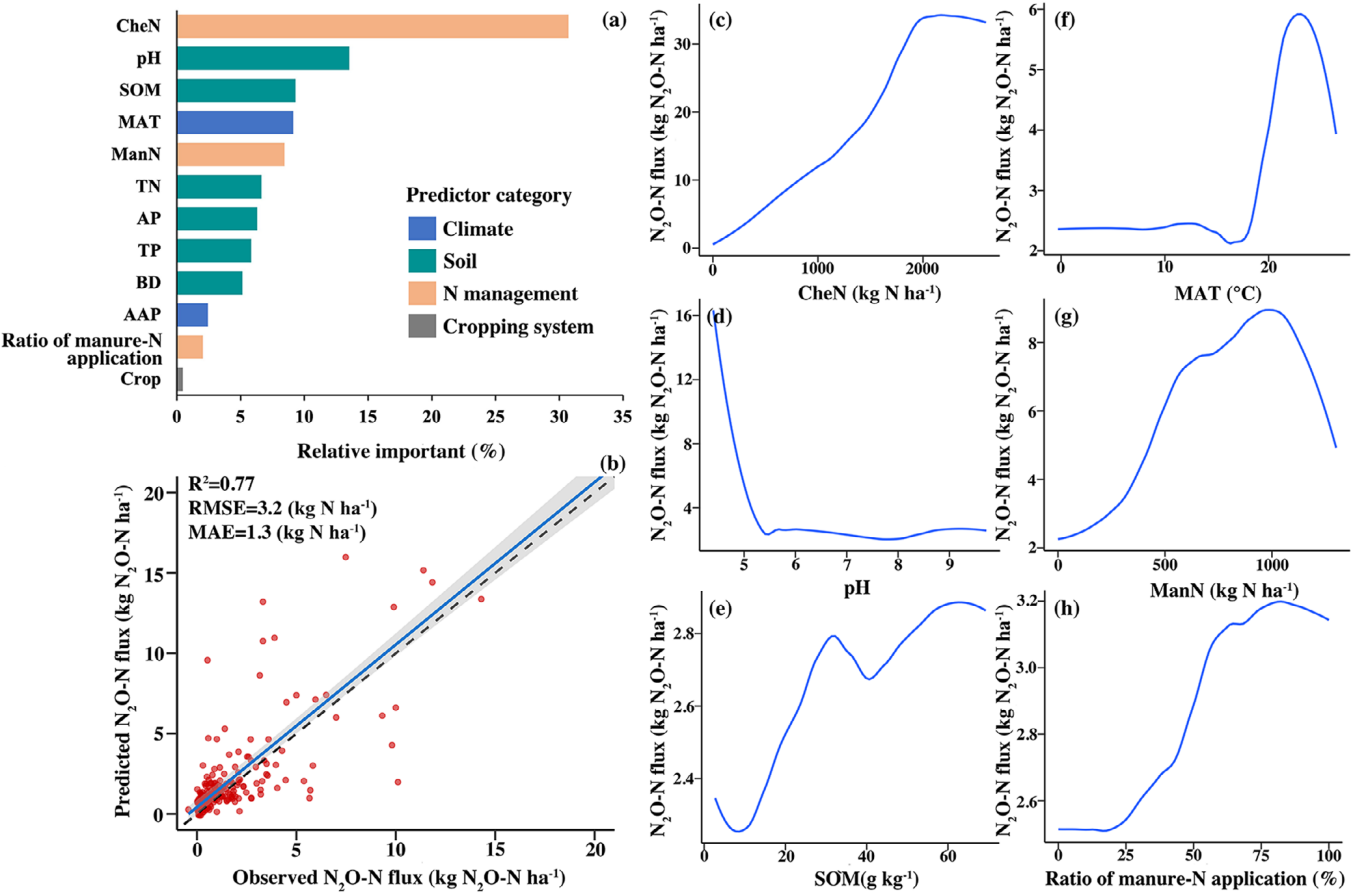
Results of the boosted regression tree model predicting N_2_O‐N fluxes in Chinese croplands. (a) Relative importance of factors in the N_2_O‐N flux model. (b) Observed versus predicted N_2_O‐N flux. The dashed line is the 1:1 line; the blue line is the regression line between observed and predicted values. Regression coefficients of determination (*R* ^2^); root mean square error (RMSE); mean absolute error (MAE). (c–h) N_2_O‐N flux response to CheN, pH, SOM, MAT, ManN and Ratio of manure‐N application, respectively. Curves represent LOESS‐smoothed partial dependence plots (PDPs) derived from the fitted boosted tree model. Chemical‐N input (CheN); pH value (pH); soil organic matter (SOM); annual mean air temperature (MAT); manure‐N input (ManN); soil total nitrogen (TN); soil available phosphorus (AP); soil total phosphorus (TP); soil bulk density (BD); annual accumulated precipitation (AAP); the ratio of manure‐N to total N application (ratio of manure‐N application) and cropping system (Crop).

The boosted regression tree model identified CheN, pH, SOM, MAT, and ManN as the five most important factors predicting cropland N_2_O‐N fluxes across China, together accounting for 71.1% of the total relative influence (Figure 3a). Their effects on N_2_O‐N flux were distinct and generally non‐linear, as shown by the PDPs (Figure 3c–h). Overall, the model explained 0.77 of the variance in the observed N_2_O‐N fluxes, with an RMSE of 3.2 kg N_2_O‐N ha^−1^ and an MAE of 1.3 kg N_2_O‐N ha^−1^ (Figure 3b and Table S1).

### 3.2 Hotspots and Spatial Distribution Trends of N_2_O‐N Loss

**FIGURE 4:**
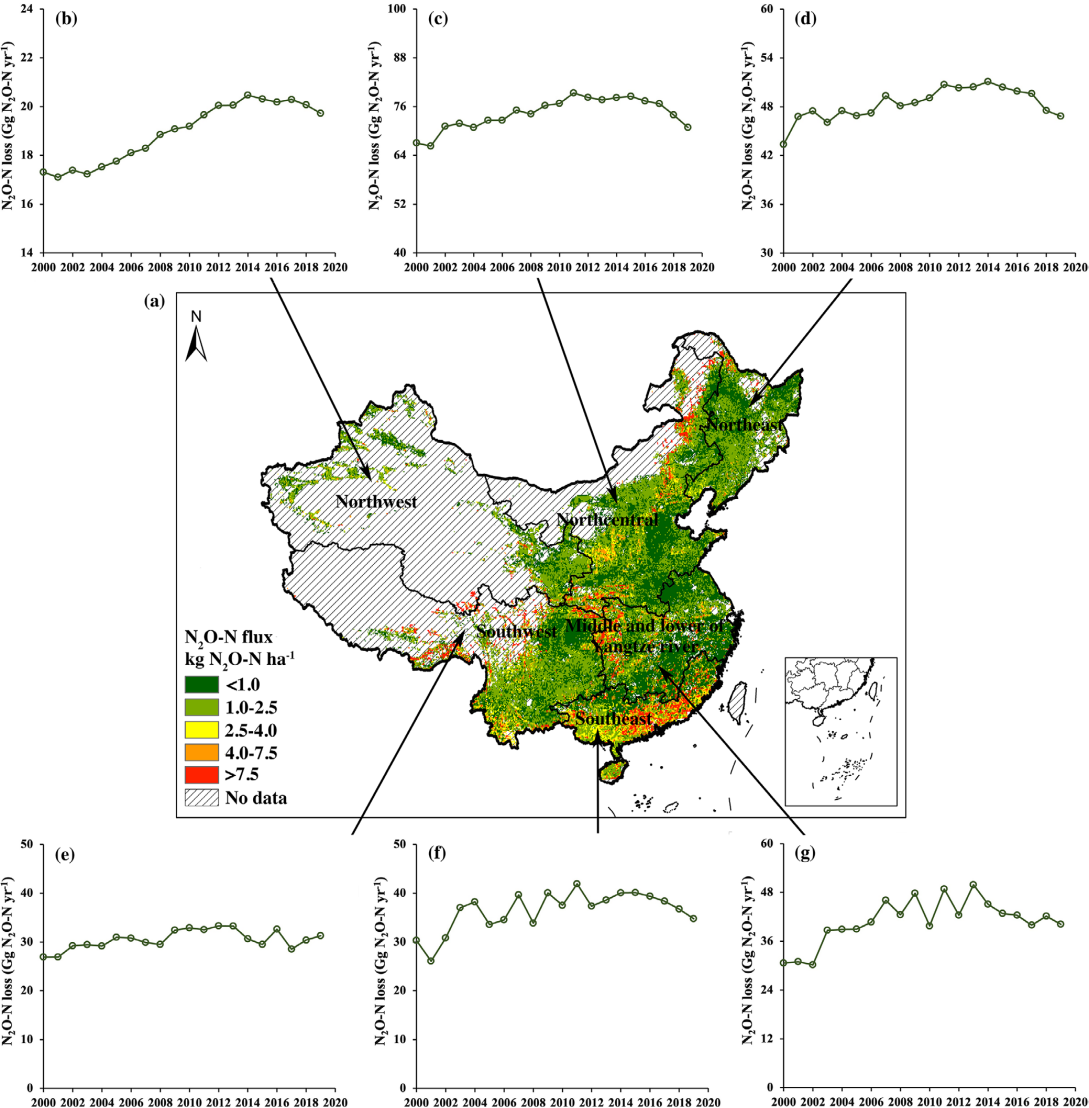
Spatial and annual distribution of annual N_2_O‐N loss in China's six agronomic regions between 2000 and 2019. (a) Geographic distribution of N_2_O‐N fluxes in Chinese cropland in 2019 at a resolution of 5 arcminutes. (b–g) Changes in N_2_O‐N losses in different regions: (b) Northwest China, (c) Northcentral China, (d) Northeast China, (e) Southwest China, (f) Southeast China and (g) Middle and Lower Yangtze River region. Note the different y‐axis scales. Map lines delineate study areas and do not necessarily depict accepted national boundaries.

The selected boosted tree model was used to simulate the spatial and interannual patterns of N_2_O‐N loss across Chinese croplands from 2000 to 2019 (Figure 4). In 2019, the nationwide N_2_O‐N flux was estimated at 1.1 ± 4.5 (median ± standard deviation) kg N_2_O‐N ha^−1^, with hotspots primarily located in southeastern China (Figure 4a). The 10 provinces with the highest N_2_O‐N losses were observed in Heilongjiang, Guangdong, Inner Mongolia, Shandong, Jilin, Hubei, Henan, Guangxi, Yunnan, and Hebei, collectively accounting for 58.8% of the total cropland N_2_O‐N loss in China (Figure 4a). From 2000 to 2019, the regional contributions to national N_2_O‐N loss remained relatively stable. The largest share was primarily from the Northcentral region (Figure 4c), while the Northwest consistently contributed the smallest share (Figure 4b). At the national scale, cropland N_2_O‐N loss increased by 49.9 Gg N_2_O‐N during 2000–2014, but then gradually declined by 21.8 Gg N_2_O‐N during 2014–2019. A similar trend was observed across most regions, with regional N_2_O‐N losses initially increasing and then declining during the same periods (Figure 4b–g).

### 3.3 Drivers of N_2_O‐N Loss Trends

**FIGURE 5:**
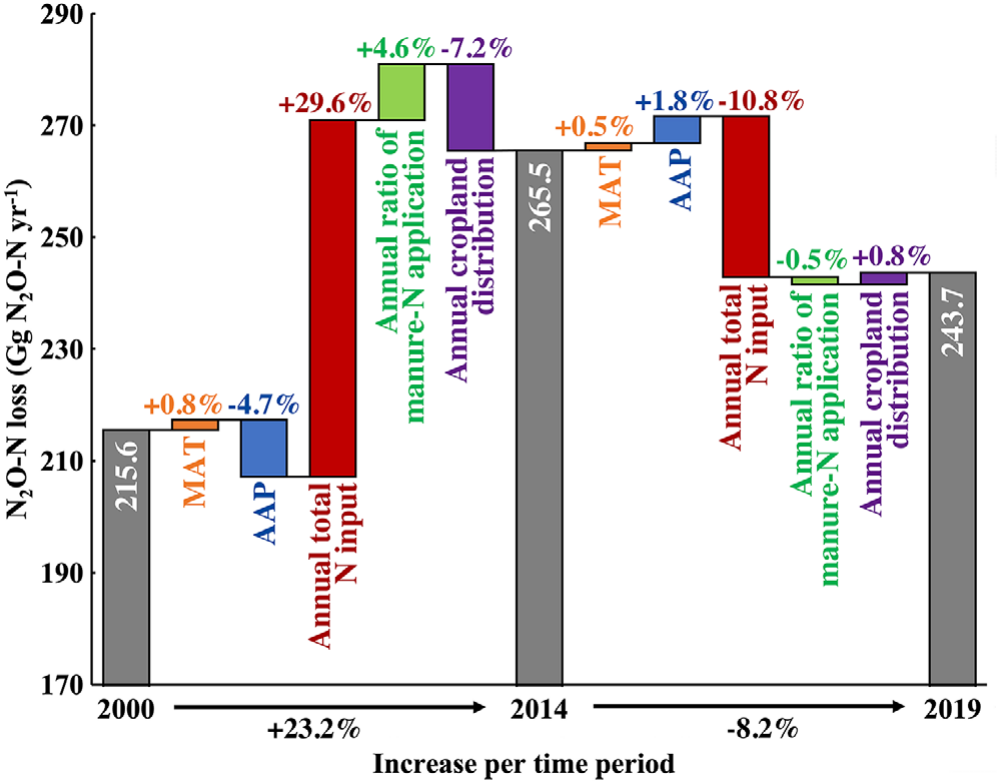
Contributions of drivers to changes in China's N_2_O‐N loss during 2000–2014 and 2014–2019. The length of each bar reflects the contribution of each driver to changes in N_2_O‐N losses during the corresponding period. Annual mean air temperature (MAT); annual accumulated precipitation (AAP); annual total N (the sum of chemical‐N and manure‐N) input (annual total N input); and the annual ratio of manure‐N to total N (the sum of chemical‐N and manure‐N) application (annual ratio of manure‐N application).

The individual contributions of the five key factors to temporal changes in N_2_O‐N loss from Chinese croplands were assessed across two time periods (Figure 5). Overall, the national trend in cropland N_2_O‐N loss between 2000 and 2019 was dominated by annual total N input, including both chemical fertilizer and manure sources (Figure 5). Before 2014, the three most important factors contributing to increased cropland N_2_O‐N loss were annual total N input, annual cropland distribution, and AAP, accounting for 29.6%, −7.2%, and −4.7% of the total change, respectively (Figure 5). After 2014, total N_2_O‐N loss decreased mainly due to a reduction in annual total N input (Figure 5). The second strongest driver during this period was the rise in AAP, which increased N_2_O‐N losses by 1.8% (Figure 5).

### 3.4 N_2_O‐N Mitigation Benefits of Manure Substitution

**FIGURE 6:**
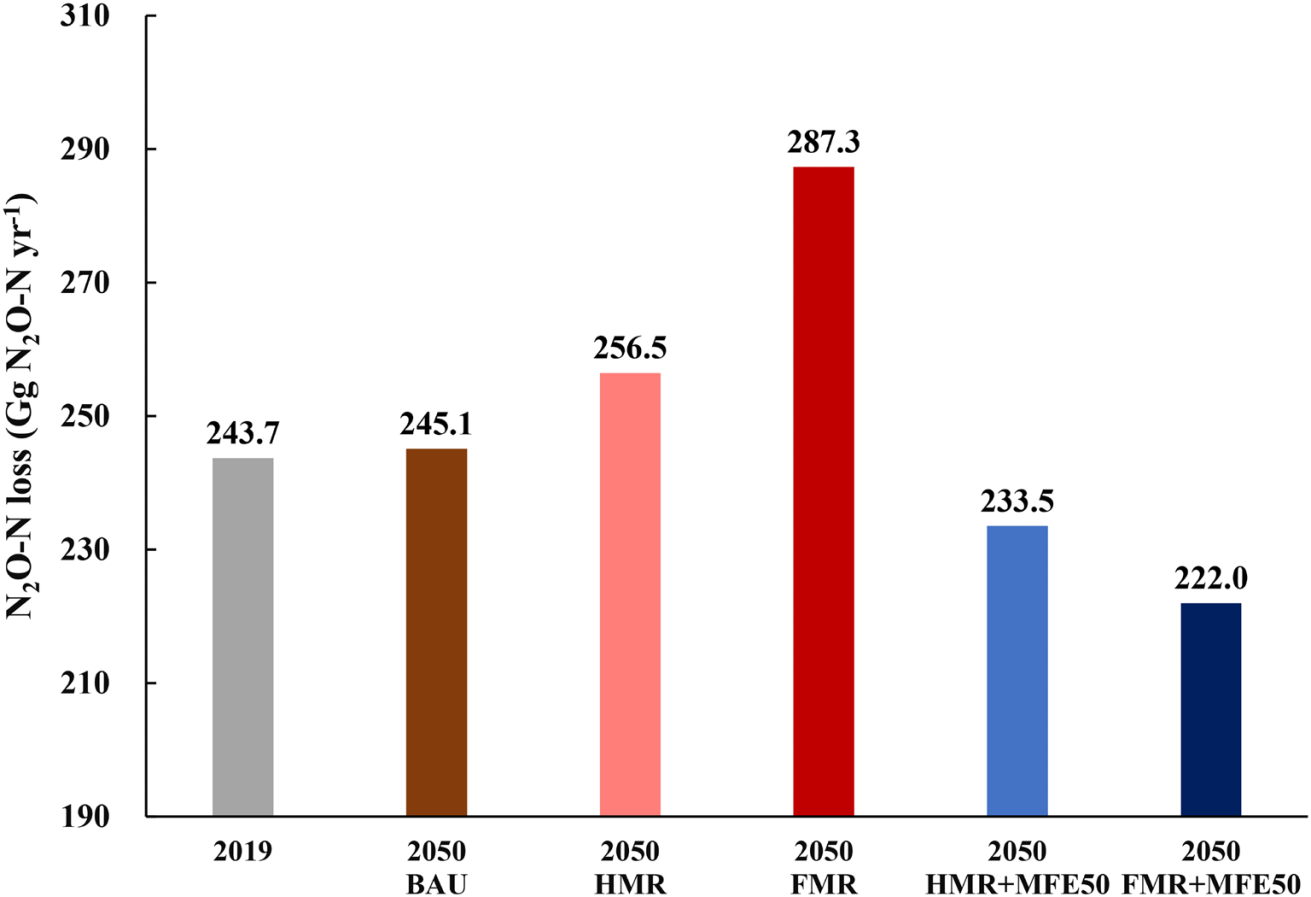
Projected national N_2_O‐N losses of fertilized croplands under different scenarios for 2050. Baseline business as usual scenario (BAU); half manure recycling scenario (HMR); full manure recycling scenario (FMR); the scenario combining half manure recycling with manure‐N fertilizer equivalency improved to 50% (HMR + MFE50); the scenario combining full manure recycling with manure‐N fertilizer equivalency improved to 50% (FMR + MFE50).

Projections for 2050 revealed a continued rise in cropland N_2_O‐N emissions in China under the BAU scenario, with total losses expected to increase by 0.60% relative to 2019 levels (Figure 6). Under the HMR and FMR scenarios, which assume 50% and 100% manure recycling without MFE improvements, national N_2_O‐N losses were projected to reach 256.5 and 287.3 Gg yr^−1^ by 2050, corresponding to increases of 5.2% and 17.9% relative to 2019 and 4.6% and 17.2% above BAU (Figure 6), respectively. In contrast, improving manure‐N use efficiency moderated these increases. Under the HMR + MFE50 scenario (half manure recycling with MFE raised to 50%), national loss was projected to decrease to 233.5 Gg yr^−1^, representing a 9.0% reduction relative to HMR (Figure 6). Under the FMR + MFE50 scenario (full manure recycling with MFE raised to 50%), the national N_2_O‐N loss was further reduced to 222.0 Gg yr^−1^, yielding the greatest mitigation potential, with a 22.7% reduction relative to FMR and a 9.4% reduction relative to BAU (Figure 6). These results highlight that future manure substitution strategies must couple high recycling rates with significant improvements in manure nutrient management; otherwise, increasing manure recycling alone may lead to 2050 emissions far exceeding BAU levels. Figures S1–S3 further provide supporting spatial maps and driver‐apportion results for these 2050 scenarios.

## 4 Discussion

### 4.1 Model Performance Evaluation

Compared to previous studies, our study incorporated three key advancements to improve the prediction of N_2_O‐N loss flux from croplands receiving both chemical fertilizers and manures in China. First, the N_2_O‐N fluxes database was compiled from 2186 study cases across 443 Chinese field studies published from January 2000 to April 2025. Accordingly, the largest and most up‐to‐date Chinese dataset was established for modeling N_2_O‐N fluxes. In contrast, the earlier mechanistic models (e.g., N_2_O_STAT, DNDC, APSIM, SPACSYS, TRIPLEX‐GHG, DLEM, VISIT, and WHCNS‐Veg) were often developed and calibrated using limited regional data or data from other countries (Table 3), which may limit their ability to represent China‐specific N_2_O‐N fluxes. Second, this study evaluated three different machine learning algorithms, with the boosted tree model identified as the best method to estimate fluxes of N_2_O‐N loss from Chinese cropland (Table S1). Unlike traditional empirical and process‐based models, the boosted tree model can better accommodate the low spatial autocorrelation of N_2_O‐N fluxes among model training samples and the non‐zonal distribution of N (chemical‐N and manure‐N) inputs and croplands, thereby potentially improving predictive accuracy and reliability (Lam et al. 2024). Specifically, our trained BRT model achieved an *R* ^2^ of 0.77 when tested against 219 field observations (Table S1), which exceeds several field‐validated *R* ^2^ values reported in previous studies for DNDC (0.40), APSIM (0.50), SPACSYS (0.41) and VISIT (0.44) (Chang et al. 2024; Ito et al. 2018; Li et al. 2021; Zhang, Zhang, et al. 2016). Third, our modeling framework explicitly distinguished between chemical‐N and manure‐N components and was the first to incorporate the ratio of manure‐N application as a key predictive variable, in combination with environmental covariates and N_2_O‐N flux measurements across a wide range of fertilization regimes (e.g., chemical‐only, manure‐only, and mixed applications with varying substitution ratios) (Table 3). This design enabled the selected boosted regression tree model, as a data‐driven statistical approach (Lam et al. 2024), to implicitly learn N_2_O‐N responses to different N‐source combinations and directly predict overall N_2_O‐N fluxes under mixed fertilization, unlike previous approaches that have computed N_2_O‐N fluxes from chemical‐N and manure‐N independently and then summed the components (Aneja et al. 2019; Bu et al. 2024; Xu et al. 2020). As a result, our integrated formulation may better capture source‐interaction effects under mixed fertilization (e.g., mineralization timing, carbon availability, soil property and microbial activity) (Xu et al. 2021; Zhang et al. 2020), thereby allowing a more precise assessment of manure amendment effects on N_2_O‐N fluxes under mixed fertilization conditions. Taken together, these three improvements contributed to a more scientifically robust and credible estimation of N_2_O‐N fluxes.

**TABLE 3:** A comparison of model characteristics in simulating N_2_O‐N loss rate from mixed chemical fertilizer and manure amendments in China.

Model	Nitrification	Denitrification	Method	References
This model	*f*(*T*_air_, P, TN, TP, AP, pH, SOM, BD, *N*_input_, *N*_type_, *N*_ratio_, Crop)		Nonlinear function‐Stochastic gradient boosting model	This study
SRMN	*f*(*T*_air_, P, TN, pH, SOC, BD, *S*_clay_, SWC *N*_input_, *N*_type_, Crop)		Nonlinear function‐Bayesian recursive regression tree model	Shang et al. (2019); Zhou, Shang, et al. (2015)
N_2_O_STAT	*f*(*T*_soil_, pH, SWC, *N*_input_, *N*_type_)		Linear function‐Multiple linear regression model	Aneja et al. (2019)
DNDC	*f*(*T*_soil_, pH, SWC, *S*_NH4_)	*f*(*T*_soil_, pH, SWC, DOC, *S*_NO3_)	Process‐based model	Chang et al. (2024), Deng et al. (2013), Gao et al. (2014), Gou et al. (1999), Jiang et al. (2023), Ke et al. (2022), Li et al. (2001), Li et al. (2010), Qiu et al. (2009), Tian, Niu, et al. (2018), Wang et al. (2011, Wang, Tao, et al. 2022), Yang et al. (2021), Yin et al. (2020), Zhang et al. (2014, 2019, Zhang et al. 2020), Zhao et al. (2020), Zhu et al. (2019)
APSIM	*f*(*T*_soil_, pH, SWC, *S*_NH4_)	*f*(*T*_soil_, SWC, DOC, *S*_NO3_)	Process‐based model	Li et al. (2021, 2016), Wang et al. (2018)
SPACSYS	*f*(*T*_soil_ SWC, S_NH4_, S_NO3_)	*f*(*T*_soil_, SWC, S_NO3_)	Process‐based model	Wang, Liu, et al. (2022), Zhang et al. (2016)
TRIPLEX‐GHG	*f*(*T*_soil_, pH, SWC, *S*_NH4_)	*f*(*T*_soil_, pH, DOC, *S*_NO3_)	Process‐based model	Song et al. (2023)
DLEM	*f*(*T*_soil_, SWC, *S*_NH4_)	*f*(*T*_soil_, *S*_clay_, *S*_rh_, S_NO3_)	Process‐based model	Tian et al. (2017), Xu et al. (2017)
VISIT	*f*(*T*_soil_, pH, SWC, *S*_NH4_)	*f*(SWC, *S*_rh_, *S*_NO3_)	Process‐based model	Ito et al. (2018)
WHCNS‐Veg	*f*(*T*_soil_, pH, SWC, *S*_NH4_)	*f*(SWC, *S*_rh_, *S*_NO3_)	Process‐based model	Li et al. (2022), Zheng et al. (2024)
PKU‐N_2_O	*f*(*N*_input_)		Empirical EF model	Xu et al. (2020), Zhou et al. (2014)
NUFER	*f*(*N*_input_)		Empirical EF model	Ma et al. (2014)
IAP‐N	*f*(*N*_input_)		Empirical EF model	Zheng et al. (2008)
STIRPAT	*f*(*N*_input_)		Empirical EF model	Bu et al. (2024)
CHEN	*f*(*N*_input_)		Empirical EF model	Luo et al. (2019)

*Note:* Annual mean air temperature (*T*_air_); annual mean soil temperature (*T*_soil_); annual accumulated precipitation (P); soil total nitrogen (TN); soil total phosphorus (TP); soil available phosphorus (AP); soil pH value (pH); soil organic matter (SOM); soil organic carbon (SOC); soil dissolved organic carbon (DOC); soil bulk destiny (BD); soil clay content (*S*_clay_); soil water content (SWC); soil heterogeneous respiration (*S*_rh_); soil NH_4_^+^ content (*S*_NH4_); soil NO_3_^−^ content (*S*_NO3_); total *N* (the sum of chemical‐N and manure‐N) input (*N*_input_); N fertilizer type (*N*_type_); the ratio of manure‐N to total N (the sum of chemical‐N and manure‐N) application (*N*_ratio_); crop type (Crop).

**FIGURE 7:**
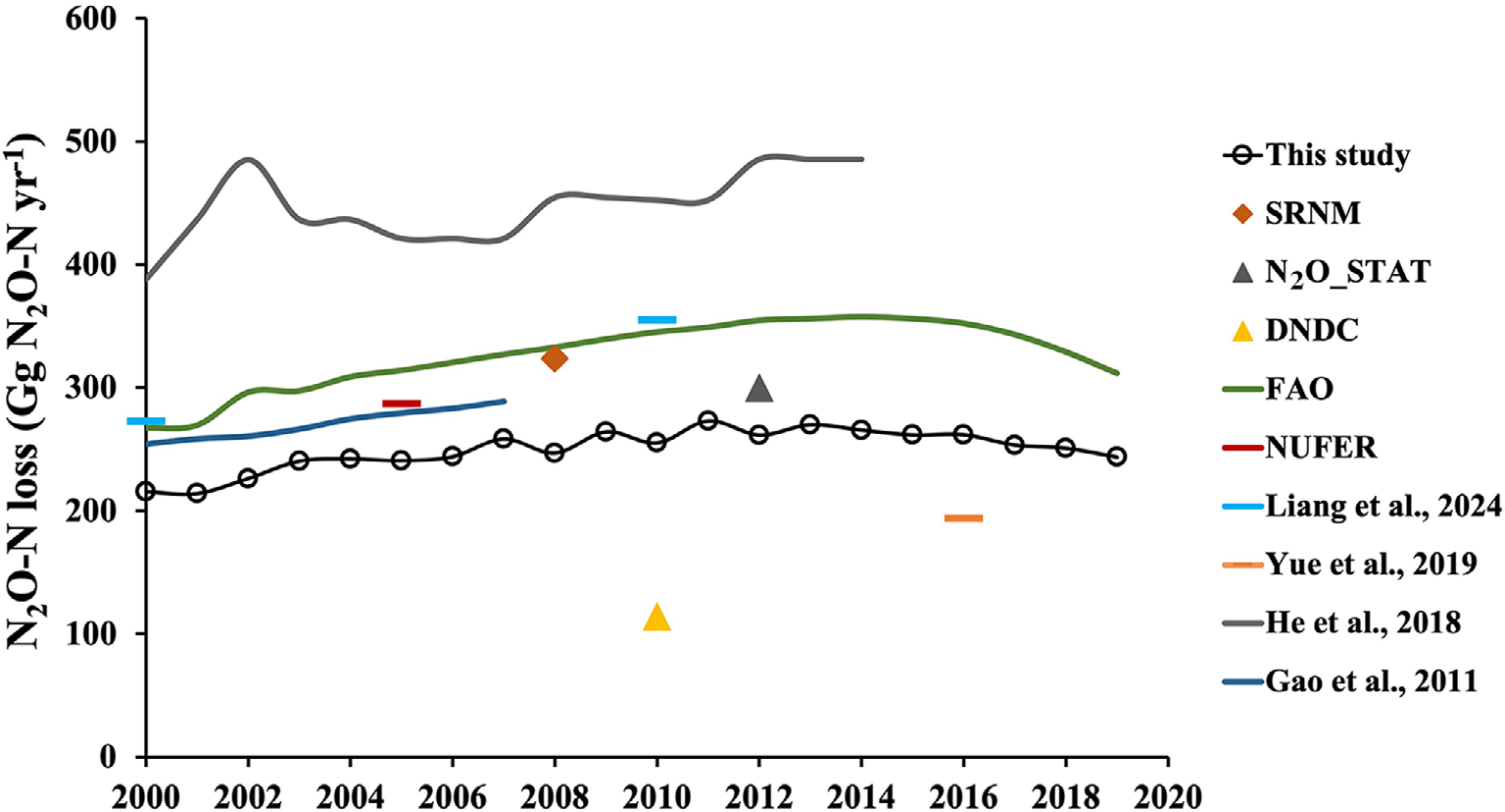
Estimates of national cropland annual N_2_O‐N losses for 2000–2019 using different methods. Examples of N_2_O‐N emission estimates are presented here. We estimated emissions by using models from SRNM, N_2_O_STAT, DNDC, and NUFER. We also present estimates from FAO, Liang et al. (2024), Yue et al. (2019), He et al. (2018), and Gao et al. (2011).

Hence, national‐scale loss of N_2_O‐N was further quantified in this study. From 2000 to 2019, the average N_2_O‐N loss across croplands in China was estimated at 249.5 Gg N_2_O‐N per year, which was close to projections from two statistical models (e.g., SRNM and N_2_O_STAT) but lower than the values reported by the majority studies using empirical EFs (Figure 7). The discrepancies between our estimates and other EF‐based inventories can be attributed to the differences in adopted EF values, methodologies, and high‐resolution activity data. To strengthen the comparison with established EF‐based inventories, we further summarized the dataset‐derived EFs from our compiled field measurements (Table S2) and compared them with EFs used in those empirical frameworks (Table S3). Overall, the EFs in our largest and most up‐to‐date dataset (paddy: 0.2%; upland: 0.5%) are generally lower than several widely used default EF values (paddy: 0.3%–1.6%; upland: 0.7%–1.6%) (Table S3), which helps explain why Chinese croplands exhibited lower N_2_O‐N emission potentials in this study compared with many EF‐based inventories (Figure 7). This highlights that accurate N_2_O‐N estimates across China require continual updating and expansion of field measurement datasets. Moreover, a more robust machine learning approach was applied to derive N_2_O‐N estimates instead of relying on cross‐site average EFs generated from limited experimental conditions, which may overestimate the N_2_O‐N losses at certain sites. Furthermore, rather than disaggregating historical county‐level chemical‐N/manure‐N statistics (e.g., statistical reports and yearbooks) to gridded cropland via spatial area allocation (Shang et al. 2019; Zhou, Shang, et al. 2015), which may smooth sub‐county heterogeneity and bias N input rates in certain cells (Wang, Zhou, et al. 2020), we used grid‐level cropland N input rates (g N m^−2^ cropland) from the HaNi dataset (Tian et al. 2022). This dataset preserves fine‐scale spatial variability and is well suited for national‐scale assessment, enabling more accurate estimates of N_2_O‐N losses and their temporal changes.

### 4.2 Spatial Pattern and Annual Variation of N_2_O‐N Loss

Wang, Liu, et al. (2022), Yang et al. (2021), Ito et al. (2018) and Tian et al. (2015) reported spatial annual terrestrial N_2_O‐N fluxes in China using the process‐based DNDC, DLEM and VISIT models, and their estimates of regional cropland N_2_O‐N fluxes were comparable to those simulated in our study, e.g., high fluxes in parts of the South and low fluxes in interior drylands and the North (Figure 4a). Simulated N_2_O‐N fluxes in 2019 were higher in the southeastern region primarily because it has the most acidic soils (median pH = 5.2) and the warmest climate (median MAT = 21.4°C) across China (Table S2). Low‐pH may enhance the inhibition of functional N_2_O reductase during denitrification, resulting in a greater mole fraction of N_2_O/(N_2_O + N_2_) (Huang et al. 2018; Mei et al. 2018; Samad et al. 2016). Additionally, the warmer temperatures in Southeast China enhance microbial turnover of substrates required for nitrification and denitrification (Knorr et al. 2005), and promote the formation of more anoxic microsites for denitrification‐driven N_2_O emissions due to increased microbial respiration (Kurganova et al. 2012), ultimately leading to higher N_2_O‐N fluxes (Song et al. 2018). Consistent with these mechanisms, our PDP analysis indicated non‐linear responses of cropland N_2_O‐N fluxes to soil pH and MAT in China, with higher fluxes under strongly acidic conditions and a marked increase around ~20°C (Figure 3d,f). In contrast, N_2_O‐N fluxes in the Middle and lower of Yangtze river region were the lowest in 2019 (Figure 4a), where the largest area of paddy fields is located nationwide (Xu et al. 2018). Paddy soils generally exhibit lower N_2_O‐N fluxes than upland soils (Mei et al. 2018; Zhang et al. 2020). Submerged paddy soils are typically dominated by anaerobic conditions, which can limit nitrification and promote the conversion of produced N_2_O‐N to N_2_, particularly following manure additions (Zhou, Zhu, et al. 2015).

Over the past two decades (2000–2019), the Northcentral consistently experienced the greatest N_2_O‐N losses nationally (Figure 4c). This predominantly flat region has become one of China's most intensive centers for vegetables (Wang, Liu, et al. 2022), arable crop and livestock production (Liu et al. 2020; Zhou et al. 2014), and is associated with the largest sown area (Yu et al. 2022) and excessive applications of chemical‐N and manure‐N (NBS 2024), resulting in elevated N_2_O‐N loss. In contrast, although Southwest China is also an important agricultural production region, its N_2_O‐N losses remained at a relatively low level from 2000 to 2019 (Figure 4b). On the one hand, dry climatic constraints, degraded soils and complex topography have limited cropland expansion (Wang et al. 2021). On the other hand, Southwest China has the lowest SOM content (median SOM = 10.9 g kg^−1^) across China (Table S2), which is unfavorable for N_2_O‐N emissions. Poor SOM content inhibits soil microbial activities, thereby reducing N transformations involved in N_2_O‐N production (e.g., nitrification and denitrification) (Harrison‐Kirk et al. 2013). Consistently, the PDP results also indicated that lower SOM was generally associated with lower N_2_O‐N fluxes (Figure 3e). Therefore, promoting agricultural development in Northwest China, such as through the advanced cropping technologies, may help alleviate the pressure of high N_2_O‐N losses in other parts of China.

Cropland N_2_O‐N losses increased by 23.2% between 2000 and 2014, followed by an 8.2% decline from 2014 to 2019 (Figure 5). The annual total N (both chemical‐N and manure‐N) input was the most important factor influencing national N_2_O‐N loss (Figure 5). Between 2000 and 2014, N_2_O‐N losses increased steadily, reflecting the rapid expansion of agricultural production in China, largely driven by excessive chemical fertilizer use and improper manure application (Sun et al. 2020). The subsequent decline observed between 2014 and 2019 was primarily attributed to recent mitigation efforts for China to discourage agricultural pollution (Liu et al. 2020), such as lowering N inputs (via chemical‐N and manure‐N) and improving NUE. These efforts included the promotion of agricultural technologies (e.g., water‐saving irrigation, straw recycling and household biogas) (Wang, Liu, et al. 2022) and were supported by national policies (e.g., “Soil Testing and Formula Fertilization” (2005), and “Zero Increase Action Plan” (Wang et al. 2015)) (Wei et al. 2021), which together have resulted in less N being applied to croplands via N chemical fertilizers and manures in 2019 compared to 2014 (FAOSTAT 2024).

The gridded median annual ratio of manure‐N application declined from 13.9% in 2000 to 10.8% in 2014, contributing 4.6% to the increase in N_2_O‐N losses (Figure 5). This decline was primarily due to the undervaluation of the manure nutrient content (Sun et al. 2020). Since 2014, national N_2_O‐N losses decreased by 0.5% (Figure 5), coinciding with a slight increase in the annual manure‐N application ratio from 10.8% to 11.2% during 2014–2019. This change reflected the positive impacts of policy interventions promoting manure nutrient recycling (e.g., *Regulations on the Prevention and Control of Pollution from Large scale Livestock and Poultry Farming* (2014), *Policy Guidance on Accelerating the Resource Utilization of Animal Manure* (2017), and *Guiding Opinions on Promoting the Land Application of Livestock Manure and Strengthening the Pollution Control According to Law* (2019)) and the practical implementation of manure utilization technologies (e.g., covered compost, anaerobic digestion and slurry injection) (Wei et al. 2021). Taken together, these two periods in Figure 5 showed that a higher manure‐N application ratio tends to contribute to N_2_O‐N mitigation. This suggests that increasing manure substitution may offer an important opportunity for mitigating N_2_O‐N losses. This effect may be attributed to (i) lower total N inputs as a result of less chemical‐N use, (ii) the additional mineralization required for manure compared to chemical fertilizers (Wang et al. 2015), which releases available N to match crop demand and reduce excess substrates for nitrification and denitrification, (iii) the promotion of complete denitrification (e.g., enhanced conversion of N_2_O to N_2_) following the addition of available organic carbon from manure, which stimulates microbial respiration, increases O_2_ consumption, and creates more anaerobic microsites (Dalal et al. 2010; Das and Adhya 2014; Sanchez‐Martin et al. 2010; Xia et al. 2018), and (iv) the accelerated microbial immobilization of NH_4_^+^ and NO_3_^−^ induced by manure carbon input (Bhattacharyya et al. 2013; Wang et al. 2015; Yue et al. 2019).

### 4.3 Implications and Recommendations

While statistical assessments and visual comparisons supported the reliability of our estimates, several potential limitations should be acknowledged: (i) Our statistical approaches are restricted to parameters available in published databases and field measurement literature. Thus, our models were unable to fully incorporate variation in agricultural practices due to limited data availability. Previous research has demonstrated that cropland N_2_O‐N losses are influenced by *N* application practices (e.g., timing, and methods) (Xia et al. 2020), as well as irrigation regimes (Huang et al. 2024; Zhang, Zhang, et al. 2024). Hence, we advocate for more comprehensive reporting of agricultural management details in future studies. (ii) Our estimates specifically focus on fertilizer‐induced N_2_O‐N emissions to emphasize the direct impact of manure substitution strategies, and therefore do not include contributions from other N sources, such as human excreta (Luo et al. 2019), crop residues (Gao et al. 2011; Luo et al. 2019), irrigation‐N (He et al. 2018), and atmospheric‐N deposition (He et al. 2018). However, these sources play important roles in shaping the spatial and temporal patterns of cropland N_2_O‐N emissions at the national scale (He et al. 2018). Therefore, we recommend that future research incorporate these additional sources to enhance the completeness of national N_2_O‐N budgets and support the development of more comprehensive mitigation strategies. (iii) All original N_2_O‐N measurements utilized in this study were obtained through chamber‐based methods, and the uncertainty associated with estimating cumulative N_2_O‐N losses at the field scale under this approach is unlikely to be less than ±50% (Wang and Lu 2020; Wang, Yao, et al. 2020). Peak N_2_O‐N fluxes may be missed by the closed static chamber method during the growing season, particularly those occurring after fertilization, tillage, or rainfall event, thereby underestimating cumulative N_2_O‐N emissions (Li et al. 2024). Currently, a combined approach involving eddy covariance (EC) measurements and chamber methods is considered as the best solution for capturing and quantifying these peak flux events (Murphy et al. 2022; Wecking et al. 2020), which should be more widely adopted in future fieldwork to improve measurement accuracy and better support model development. (iv) The gridded soil property data from HWSD used in this study were sourced from the Second National Soil Survey of China (1979–1986), and therefore may not fully represent current soil conditions. The HaNi dataset currently provides nationwide gridded chemical‐N and manure‐N input rates only through 2019, which constrained our ability to extend the assessment of N_2_O‐N emissions to more recent years (Tian et al. 2022). Hence, we recommend that these high‐resolution gridded datasets should be continually updated, as this is essential for evaluating post‐2019 cropland N_2_O‐N emissions and for supporting the design and tracking of manure‐management policies (Ito et al. 2018).

In this study, we developed a novel modeling framework to accurately quantify N_2_O‐N fluxes under mixed fertilization conditions, which accounted for 20.1% of cropland in 2019 and are non‐negligible and increasingly relevant in China, aimed at improving the national‐scale assessments of cropland N_2_O‐N spatial distributions, temporal dynamics, and mitigation potential. It provided high‐resolution estimates of N_2_O‐N fluxes, which are useful for identifying national hotspots of cropland N_2_O‐N loss. For example, the Northcentral region consistently exhibited the highest N_2_O‐N losses between 2000 and 2019 (Figure 4c), whereas Northwest China maintained the lowest losses (Figure 4b). Moreover, over the same period, N_2_O‐N losses were mainly driven by annual total N input (Figure 5), demonstrating the importance of continued implementation of N reduction and nutrient management strategies as core pillars of greener agricultural development.

These findings are valuable for informing region‐specific abatement decisions. The historical stability in both spatial patterns and dominant drivers suggests that, in the absence of substantial policy‐driven changes in fertilization practices, the Northcentral region will likely remain an N_2_O‐N hotspot in the near future. This region should therefore be prioritized for environmental policy intervention, while Northwest China with the lowest level of losses could be encouraged to expand agricultural development (e.g., advanced cropping technologies) to help reduce national N_2_O‐pollution pressure.

Furthermore, projections for 2050 indicated that China is likely to face a continued high risk of increasing cropland N_2_O‐N losses, driven by rising food demand necessitating greater total N (chemical‐N and manure‐N) inputs, as well as the anticipated impacts of climate and cropland changes (Figures 6 and S1). However, it should be noted that despite the 50% and 100% manure recycling under the HMR and FMR scenarios, N_2_O‐N losses were still projected to rise to 256.5 and 287.3 Gg yr^−1^ by 2050, representing 5.2% and 17.9% increases compared to 2019 respectively (Figures 6, S2a and S2b). The elevated emissions under HMR or FMR stem from an increase in total N input associated with the low MFE value (30%) (Figure S3a,b). This is because manure substitution changes not only the N‐input structure but can also increase the total N‐input amount: low‐quality manure‐N contains less readily available N than chemical‐N, and additional N inputs may be required to maintain crop yields (Jensen 2013). Taken together, these results further indicated that the net mitigation effect of manure substitution, as suggested by Figure 5, may weaken or reverse if total N input increases simultaneously (Figures 6 and S3a,b). These results demonstrate for the first time that achieving full manure recycling, an ambition aligned with China's national agricultural policy targets, is insufficient to mitigate N_2_O‐N losses and may even markedly exacerbate them under current poor‐quality manure N management. Our new findings challenge the current focus on recycling rate alone and suggest that long‐term national policy should promote optimal manure management to enhance manure‐N use efficiency.

In contrast, scenarios combining full manure recycling with improved MFE (FMR + MFE50) substantially moderated the increase observed under FMR (Figures 6 and S2d). The pronounced mitigation observed under the FMR + MFE50 scenario highlights that conserving the plant‐available N content of manure (enhanced‐MFE) through better manure storage and processing techniques and adopting low‐emission manure spreading at times of crop demand, is essential for realizing the full N_2_O‐N loss mitigation potential of manure substitution, by both improving the N‐input structure and limiting compensatory increases in total N input through higher manure‐N use efficiency (Figure S3b,d). In addition, its broader co‐benefits should not be overlooked. For example, it would avoid the production and application of 9.5 Tg of chemical N fertilizer, accounting for 36.3% of agricultural N inputs under the BAU (Table 2). Additionally, the national manure substitution rate of 41.9% (Table 2) assumed in the FMR + MFE50 has been shown in multiple studies not to compromise crop yields (Ren et al. 2022; Xia et al. 2018). Nevertheless, China's current manure management still has substantial room for improvement, particularly when compared with global best practices. It is notable that a 35.2% manure recycling rate combined with a 30.0% MFE resulted in manure‐N offsetting only 5.4% of chemical‐N input in 2019 (Table 2). In contrast, for instance, in the European Union (EU‐27), manure application in 2020 reduced chemical‐N use by 0.9 Tg, accounting for 8.5% of the total (Lampkin and Padel 2022). This reduction was supported by the expansion of organically managed farmland to 15 Mha in 2020 (9.1% of EU‐27 farmland), where the synthetic fertilizers are not used and nutrient supply relies primarily on recycled organic amendments (e.g., solid manure, slurries and biogas digestate), supplemented by legumes in crop rotations (Lampkin and Padel 2022). Building on this progress, the European Commission launched the Farm‐to‐Fork (F2F) Strategy in 2020, which targets a further 20% reduction in chemical‐N use by 2030, driven by further increasing the organic share of agricultural land in the EU‐27 to 25% (European Commission 2020). At the global scale, Devault et al. (2025) estimated that recycling nutrients from underutilized manure could reduce the need for up to 49% of currently applied mineral fertilizers worldwide (Devault et al. 2025). Recently, demonstration programmes have been launched in 100 counties across China to promote manure utilization by increasing manure recycling rates and improving MFE values, and a further 200 counties are planned (Jin et al. 2020). However, achieving nationwide improvements in both manure recycling and MFE will require sustained national support, including related training and extension, technology development, manure management infrastructure, and a field‐level manure nutrient recommendation system (Chadwick et al. 2020).

Overall, this study provides a high‐resolution modeling framework to predict how developments in manure substitution will affect the magnitude and spatial distribution of N_2_O‐N losses, and highlights that future manure substitution policies should prioritize both the improved recycling rate and manure N management, while also warning of potential unintended environmental trade‐offs if improvements in manure management are not adequately addressed.

## Author Contributions

**Zhaohai Bai:** writing – review and editing, conceptualization. **Davey L. Jones:** writing – review and editing. **Shidi Ba:** writing – original draft, visualization, validation, resources, methodology, investigation, formal analysis, data curation, conceptualization.

## Funding

This work was supported by China Scholarship Council (202108130063).

## Conflicts of Interest

The authors declare no conflicts of interest.

## Supporting information


**Figure S1:** Geographic distribution of N_2_O‐N fluxes in Chinese cropland in 2050 under business‐as‐usual (BAU) scenario. Map lines delineate study areas and do not necessarily depict accepted national boundaries.
**Figure S2:** Spatial differences of N_2_O‐N fluxes in Chinese cropland in 2050 relative to the business‐as‐usual (BAU) scenario. (a) half manure recycling scenario (HMR); (b) full manure recycling scenario (FMR); (c) the scenario combining half manure recycling with manure‐N fertilizer equivalency improved to 50% (HMR + MFE50); (d) the scenario combining full manure recycling with manure‐N fertilizer equivalency improved to 50% (FMR + MFE50). Map lines delineate study areas and do not necessarily depict accepted national boundaries.
**Figure S3:** Contributions of drivers to changes in China's N_2_O‐N loss during 2019–2050 under each enhanced scenario. (a) half manure recycling scenario (HMR); (b) full manure recycling scenario (FMR); (c) the scenario combining half manure recycling with manure‐N fertilizer equivalency improved to 50% (HMR + MFE50); (d) the scenario combining full manure recycling with manure‐N fertilizer equivalency improved to 50% (FMR + MFE50). The length of each bar reflects the contribution of each driver to changes in N_2_O‐N losses during the corresponding period. Annual mean air temperature (MAT); annual accumulated precipitation (AAP); annual total *N* (the sum of chemical‐N and manure‐N) input (annual total *N* input); and the annual ratio of manure‐N to total *N* (the sum of chemical‐N and manure‐N) application (annual ratio of manure‐N application).
**Table S1:** Performance comparison of different modeling approaches.
**Table S2:** N_2_O‐N emission factors (EFs) and key characteristics of six agricultural regions in China.
**Table S3:** Comparison of N_2_O‐N emission factors (EFs) across published empirical models.
**Text S1:** Machine‐learning method description.
**Text S2:** Referenced model description.
**Text S3:** Indicator definition.
**Text S4:** The construction of gridded chemical‐N and manure‐N input rates for 2050 under five different scenarios.

## Data Availability

The data that support the findings of this study are openly available in Zenodo at https://doi.org/10.5281/zenodo.19144029. The computer code for model construction is available at https://doi.org/10.5281/zenodo.19144282.
