# Supplementary material for: Full Manure Recycling Risks an 18% Rise in China's Cropland N2O Emissions Without Improved Management
Source: Glob Chang Biol. 2026 Apr 12;32(4):e70837. doi: 10.1111/gcb.70837 (PMC13071492; doi:10.1111/gcb.70837)
Supplement: Supplementary file 1 — Figure S1: Geographic distribution of N2O‐N fluxes in Chinese cropland in 2050 under business‐as‐usual (BAU) scenario. Map lines delineate study areas and do not necessarily depict accepted national boundaries. Figure S2: Spatial differences of N2O‐N fluxes in Chinese cropland in 2050 relative to the business‐as‐usual (BAU) scenario. (a) half manure recycling scenario (HMR); (b) full manure recycling scenario (FMR); (c) the scenario combining half manure recycling with manure‐N fertilizer equivalency improved to 50% (HMR + MFE50); (d) the scenario combining full manure recycling with manure‐N fertilizer equivalency improved to 50% (FMR + MFE50). Map lines delineate study areas and do not necessarily depict accepted national boundaries. Figure S3: Contributions of drivers to changes in China's N2O‐N loss during 2019–2050 under each enhanced scenario. (a) half manure recycling scenario (HMR); (b) full manure recycling scenario (FMR); (c) the scenario combining half manure recycling with manure‐N fertilizer equivalency improved to 50% (HMR + MFE50); (d) the scenario combining full manure recycling with manure‐N fertilizer equivalency improved to 50% (FMR + MFE50). The length of each bar reflects the contribution of each driver to changes in N2O‐N losses during the corresponding period. Annual mean air temperature (MAT); annual accumulated precipitation (AAP); annual total N (the sum of chemical‐N and manure‐N) input (annual total N input); and the annual ratio of manure‐N to total N (the sum of chemical‐N and manure‐N) application (annual ratio of manure‐N application). Table S1: Performance comparison of different modeling approaches. Table S2: N2O‐N emission factors (EFs) and key characteristics of six agricultural regions in China. Table S3: Comparison of N2O‐N emission factors (EFs) across published empirical models. Text S1: Machine‐learning method description. Text S2: Referenced model description. Text S3: Indicator definition. Text S4: The construction of gridd [file GCB-32-e70837-s001.pdf]

SUPPORTING INFORMATION

Full Manure Recycling Risks an 18% Rise in China's Cropland N_2O Emissions without Improved Management

Shidi Ba [a], Zhaohai Bai [b], Lin Ma [b,c], Davey L. Jones [a], David R. Chadwick [a,*]

[a] School of Environmental and Natural Sciences, Bangor University, Bangor LL57 2UW, United Kingdom

[b] Key Laboratory of Agricultural Water Resources, Center for Agricultural Resources Research, Institute of Genetics and Developmental Biology, Chinese Academy of Sciences, 286 Huaizhong Road, Shijiazhuang 050021, China

[c] State Key Laboratory of Pollution Control and Resource Reuse, School of the Environment, Nanjing University, Nanjing 210023, China

Correspondence: David R. Chadwick (Tel: +44 1248383569; Email:d.chadwick@bangor.ac.uk)

Overview

This document provides supporting information for the article "Full Manure Recycling Risks an 18% Rise in China's Cropland N_2O Emissions without Improved Management", including descriptions of the machine-learning methods, referenced models, indicator definitions, the construction of gridded N input rates for 2050 under five scenarios, additional figures and tables that support the main results.

Supporting Text

Text S1. Machine-learning method description

Generalized linear model:

The generalized linear model is a flexible extension of ordinary linear regression, which allows for response variables with non-normal error distributions by linking the mean of the response variable to the linear predictors via a specified link function (Nelder and Wedderburn 1972). This method offers several advantages, including simplicity in implementation, interpretability of model coefficients, computational efficiency and suitability for small datasets. However, generalized linear models assume linear relationships between predictors and the response variable and may perform poorly when capturing complex nonlinear interactions or when important variables are missing or misrepresented.

Random forest model:

Random forests uses bagging for classification and regression by constructing a multitude of decision trees during the training process (Breiman 2001). This approach offers several advantages, including high predictive accuracy, low risk of overfitting, tolerance to missing values, compatibility with both categorical and continuous variables, effectiveness in handling unbalanced datasets, capability for capturing nonlinear interactions and user-friendliness. However, the method may perform poorly on very high-dimensional and sparse datasets.

Boosted regression tree (BRT) model:

The BRT is an ensemble learning method based on iterative decision tree algorithms,

which optimizes a loss function by adding trees that predict the residuals or errors of prior trees merged into the ensemble (Friedman 2001 and 2002). This approach provides several strengths, such as superior predictive performance, strong resistance to overfitting, robustness to missing data, flexibility in handling both categorical and numerical predictors, effectiveness in capturing nonlinear interactions, insensitivity to outliers by optimizing different loss functions, and no requirement for feature scaling. However, it can be computationally expensive, sensitive to noise, and intensive to parameterize.

Text S2. Referenced model description

GLOBIOM-China:

GLOBIOM-China is a bottom-up, recursive and dynamic partial equilibrium economic model that focuses on China's key land-use sectors, including crops, livestock, forestry, and bioenergy (Zhao et al. 2021). It is integrated with recent Chinese statistics and policies (Zhao et al. 2021). The agricultural product requirements are generated based on population data, gross domestic product (GDP), and equilibrium prices. Crop yields under different cropping systems are estimated using the crop biophysical model, Environmental Policy Integrated Climate (EPIC) (Balkovič et al. 2014; Williams et al. 1989). The feed demand and livestock production across various livestock production systems are derived from the RUMINANT model (Herrero et al. 2021). Trade flow projections for agricultural products are modeled bilaterally using the spatial-equilibrium approach (Takayama et al. 1984).

NUFER:

The NUFER model simulates nutrient flows in the "soil-crop-livestock-food processing-food consumption" chain for all major animal species and livestock systems in China (Ma et al. 2010). It is based on human and agricultural activities and is parameterized using detailed emission factors to evaluate nutrient losses, manure management, resource inputs, and food exports for a given year (Bai et al. 2022; Jin et al. 2020).

Text S3. Indicator definition

Manure recycling rate: Manure recycling rate is the proportion of the excreta generated by livestock that is returned to the cropland each year (%).

Manure-N fertilizer equivalency (MFE) value: The MFE expresses the fertilizer-equivalent fraction of manure-N relative to chemical-N on a plant-available N basis (%) (Jensen 2013). It is used to determine the chemical-N supplement required to maintain the same available-N supply under different manure recycling rates, thereby defining the manure substitution level. In this study, MFE was treated as a scenario parameter reflecting manure-N availability as affected by improvements in manure management (e.g., storage, processing, and spreading) prior to land application (Webb et al. 2013).

Text S4. The construction of gridded chemical-N and manure-N input rates for 2050 under five different scenarios

BAU (baseline business as usual) scenario:

The total manure-N input in the BAU was calculated using the following equation (Eq. A.1):

$$F_{\text{man,BAU}} = E_{\text{man,2050}} \times r_{\text{BAU}} \tag{A.1}$$

$F_{\text{man,BAU}}$ represents the total manure-N input (Tg N) under the BAU scenario. $E_{\text{man,2050}}$ denotes the total manure-N excretion (Tg N) in 2050, which was estimated using the NUFER model, driven by the projected livestock population from GLOBIOM-China following the SSP2 storyline (Zhao et al. 2021). The parameter r_{BAU} represents the manure recycling rate (%) under the BAU scenario, which was assumed to remain the same as 2019 (35.17%) without additional manure-management optimization measures (FAOSTAT 2024).

The gridded chemical-N and manure-N input rates of the BAU scenario in 2050 were generated by scaling the 2019 maps of chemical-N and manure-N input rates as follows Eq. (A.2–A.5):

$$R_{\text{che,BAU}} = \frac{F_{\text{che,BAU}}}{F_{\text{che,2019}}} \tag{A.2}$$

$$R_{\text{man,BAU}} = \frac{F_{\text{man,BAU}}}{F_{\text{man,2019}}} \tag{A.3}$$

$$N_{\text{che,BAU}} = N_{\text{che,2019}} \times R_{\text{che,BAU}} \tag{A.4}$$

$$N_{\text{man,BAU}} = N_{\text{man,2019}} \times R_{\text{man,BAU}} \tag{A.5}$$

where $R_{\text{che,BAU}}$ and $R_{\text{man,BAU}}$ are the unitless scaling factors for the BAU scenario. $F_{\text{che,BAU}}$ is the total chemical-N input (Tg N) under the BAU scenario, assessed using coupled projections from GLOBIOM-China and NUFER under the SSP2 storyline (Bai et al. 2022; Jin et al. 2020; Zhao et al. 2021). $F_{\text{che,2019}}$ and $F_{\text{man,2019}}$ represent the total chemical-N input and manure-N input to cropland (Tg N) in 2019, respectively, as derived from FAOSTAT (FAOSTAT 2024). $N_{\text{che,2019}}$ and $N_{\text{man,2019}}$ are the gridded chemical-N and manure-N input rates (g N m^{-2} cropland) for 2019 respectively, derived

from the Harmonized Anthropogenic N Inputs (HaNi) dataset (Tian et al. 2022). $N_{\mathrm{che,BAU}}$ and $N_{\mathrm{man,BAU}}$ are the projected chemical-N and manure-N input rates (g N m^{-2} cropland) at the grid level under the BAU scenario, respectively.

HMR (half manure recycling) and FMR (full manure recycling) scenarios:

Based on the same total available-N input as the BAU scenario, the total chemical-N and manure-N inputs under the HMR and FMR scenarios were calculated using the following equations (Eq. A.6–A.10):

$$F_{\mathrm{man,HMR}} = E_{\mathrm{man,2050}} \times r_{\mathrm{HMR}} \quad \text{(A.6)}$$

$$F_{\mathrm{man,FMR}} = E_{\mathrm{man,2050}} \times r_{\mathrm{FMR}} \quad \text{(A.7)}$$

$$TN_{\mathrm{avail}} = F_{\mathrm{man,BAU}} \times e_{\mathrm{BAU}} + F_{\mathrm{che,BAU}} \quad \text{(A.8)}$$

$$F_{\mathrm{che,HMR}} = TN_{\mathrm{avail}} - F_{\mathrm{man,HMR}} \times e_{\mathrm{HMR}} \quad \text{(A.9)}$$

$$F_{\mathrm{che,FMR}} = TN_{\mathrm{avail}} - F_{\mathrm{man,FMR}} \times e_{\mathrm{FMR}} \quad \text{(A.10)}$$

$F_{\mathrm{man,HMR}}$ and $F_{\mathrm{man,FMR}}$ represent the total manure-N input (Tg N) under HMR and FMR, respectively. r_{HMR} and r_{FMR} are manure recycling rates (%), set to 50% and 100%, representing half and full recycling of collectable manure back to cropland. TN_{avail} denotes the total available-N input (Tg N). e_{BAU}, e_{HMR} and e_{FMR} are MFE values (%), set to 30% (Bai et al. 2016; Jensen 2013). $F_{\mathrm{che},H\mathrm{MR}}$ and $F_{\mathrm{che,FMR}}$ are the total chemical-N input (Tg N) under the HMR and FMR scenarios.

Furthermore, the grid-level chemical-N and manure-N input rates for the HMR and FMR scenarios were determined using the following equations (Eq. A.11–A.18):

$$R_{\mathrm{che,HMR}} = \frac{F_{\mathrm{che,HMR}}}{F_{\mathrm{che,2019}}} \quad \text{(A.11)}$$

$$R_{\mathrm{man,HMR}} = \frac{F_{\mathrm{man,HMR}}}{F_{\mathrm{man,2019}}} \quad \text{(A.12)}$$

$$R_{\text{che,FMR}} = \frac{F_{\text{che,FMR}}}{F_{\text{che,2019}}} \tag{A.13}$$

$$R_{\text{man,FMR}} = \frac{F_{\text{man,FMR}}}{F_{\text{man,2019}}} \tag{A.14}$$

$$N_{\text{che,HMR}} = N_{\text{che,2019}} \times R_{\text{che,}H\text{MR}} \tag{A.15}$$

$$N_{\text{man,HMR}} = N_{\text{man,2019}} \times R_{\text{man,HMR}} \tag{A.16}$$

$$N_{\text{che,FMR}} = N_{\text{che,2019}} \times R_{\text{che,FMR}} \tag{A.17}$$

$$N_{\text{man,FMR}} = N_{\text{man,2019}} \times R_{\text{man,FMR}} \tag{A.18}$$

where $R_{\text{che,HMR}}$, $R_{\text{man,HMR}}$, $R_{\text{che,FMR}}$, and $R_{\text{man,FMR}}$ are the scaling factors (unitless), and $N_{\text{che,}H\text{MR}}$, $N_{man,H\text{MR}}$, $N_{\text{che,}F\text{MR}}$ and $N_{man,F\text{MR}}$ represent the gridded chemical-N or manure-N input rate (g N m^{-2} cropland) under the corresponding scenarios.

HMR+MFE50 (half manure recycling with 50% manure fertilizer equivalency) and FMR+MFE50 (full manure recycling with 50% manure fertilizer equivalency) scenarios:

These represent further optimizations of the HMR and FMR scenarios, with same total available-N input as in the BAU. The total chemical-N and manure-N inputs in the HMR+MFE50 and FMR+MFE50 scenarios were calculated using the following equations (Eq. A.19–A.22):

$$F_{\text{man,HMR+MFE50}} = F_{\text{man,HMR}} \tag{A.19}$$

$$F_{\text{man,FMR+MFE50}} = F_{\text{man,FMR}} \tag{A.20}$$

$$F_{\text{che,HMR+MFE50}} = TN_{\text{avail}} - F_{\text{man,HMR+MFE50}} \times e_{\text{HMR+MFE50}} \tag{A.21}$$

$$F_{\text{che,FMR+MFE50}} = TN_{\text{avail}} - F_{\text{man,FMR+MFE50}} \times e_{\text{FMR+MFE50}} \tag{A.22}$$

$F_{\text{man,HMR+MFE50}}$ and $F_{\text{man,FMR+MFE50}}$ mean the total manure-N input (Tg N) under

the HMR+MFE50 and FMR+MFE50 scenarios, respectively. $e_{\mathrm{HMR+MFE50}}$ and $e_{\mathrm{FMR+MFE50}}$ are the MFE values (%), set to 50%. $F_{\mathrm{che,HMR+MFE50}}$ and $F_{\mathrm{che,FMR+MFE50}}$ are the total chemical-N input (Tg N) under the HMR+MFE50 and FMR+MFE50 scenarios, respectively.

Moreover, the HMR+MFE50 and FMR+MFE50 scenarios' grid-level chemical-N and manure-N input rates were derived from Eq. A.23–A.30.

$$R_{\mathrm{che,HMR+MFE50}} = \frac{F_{\mathrm{che,HMR+MFE50}}}{F_{\mathrm{che,2019}}} \tag{A.23}$$

$$R_{\mathrm{man,HMR+MFE50}} = \frac{F_{\mathrm{man,HMR+MFE50}}}{F_{\mathrm{man,2019}}} \tag{A.24}$$

$$R_{\mathrm{che,FMR+MFE50}} = \frac{F_{\mathrm{che,FMR+MFE50}}}{F_{\mathrm{che,2019}}} \tag{A.25}$$

$$R_{\mathrm{man,FMR+MFE50}} = \frac{F_{\mathrm{man,FMR+MFE50}}}{F_{\mathrm{man,2019}}} \tag{A.26}$$

$$N_{\mathrm{che,HMR+MFE50}} = N_{\mathrm{che,2019}} \times R_{\mathrm{che,HMR+MFE50}} \tag{A.27}$$

$$N_{\mathrm{man,HMR+MFE50}} = N_{\mathrm{man,2019}} \times R_{\mathrm{man,HMR+MFE50}} \tag{A.28}$$

$$N_{\mathrm{che,FMR+MFE50}} = N_{\mathrm{che,2019}} \times R_{\mathrm{che,FMR+MFE50}} \tag{A.29}$$

$$N_{\mathrm{man,FMR+MFE50}} = N_{\mathrm{man,2019}} \times R_{\mathrm{man,FMR+MFE50}} \tag{A.30}$$

where $R_{\mathrm{che,HMR+MFE50}}$, $R_{\mathrm{man,HMR+MFE50}}$, $R_{\mathrm{che,FMR+MFE50}}$ and $R_{\mathrm{man,FMR+MFE50}}$ are the regulation ratios (unitless), and $N_{\mathrm{che,HMR+MFE50}}$, $N_{\mathrm{man,HMR+MFE50}}$ $N_{\mathrm{che,FMR+MFE50}}$ and $N_{\mathrm{man,FMR+MFE50}}$ represent the gridded chemical-N or manure-N input rate (g N m^{-2} cropland) under the corresponding scenarios.

Supporting Figure

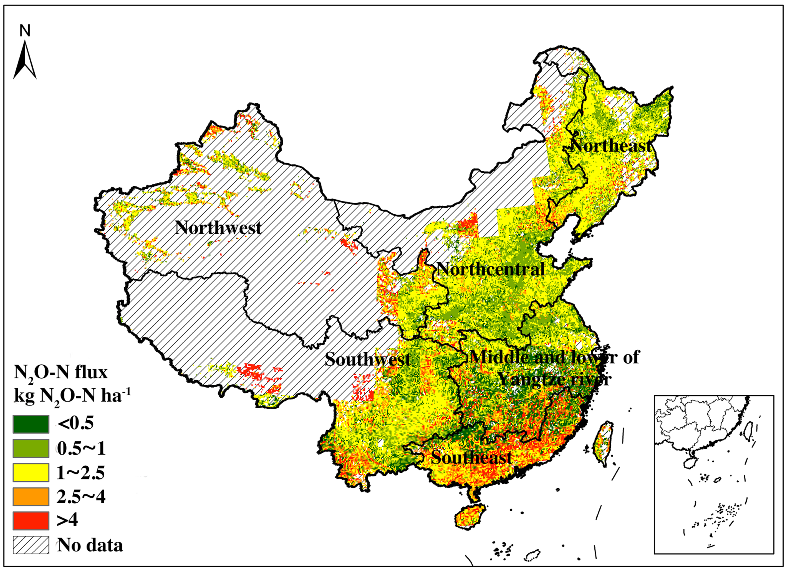

Figure S1. Geographic distribution of N_2O-N fluxes in Chinese cropland in 2050 under business-as-usual (BAU) scenario. Map lines delineate study areas and do not necessarily depict accepted national boundaries.

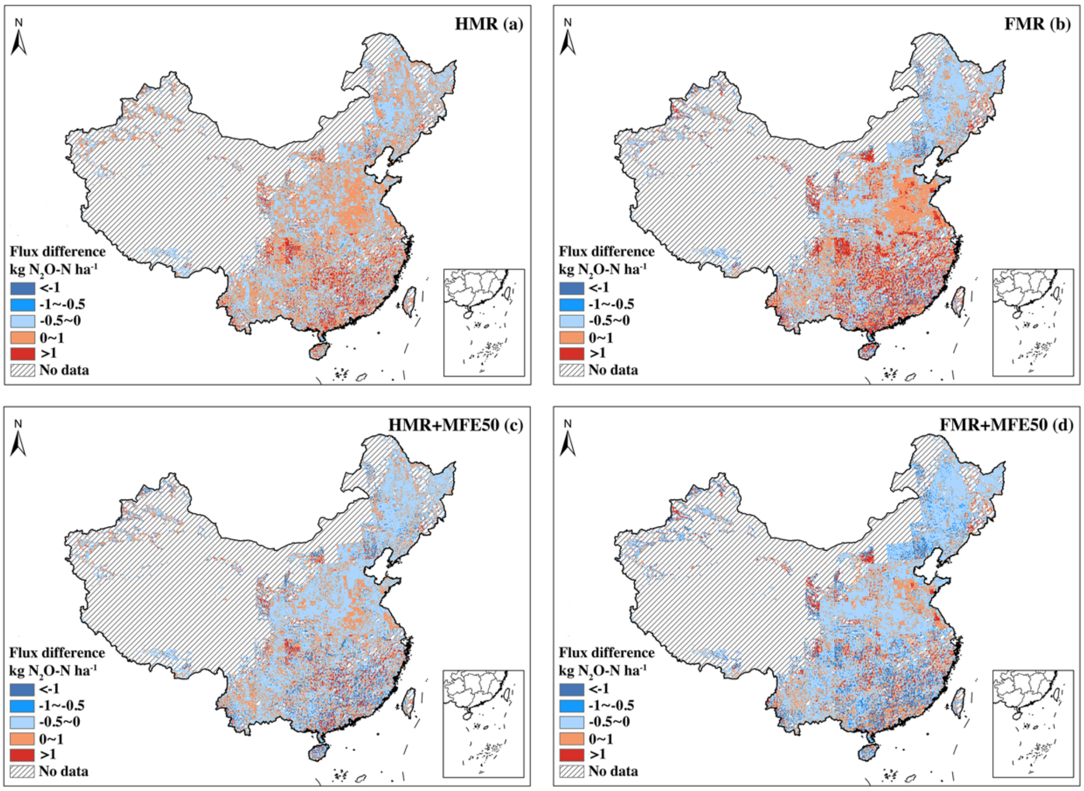

Figure S2. Spatial differences of N_2O-N fluxes in Chinese cropland in 2050 relative to the business-as-usual (BAU) scenario. (a) half manure recycling scenario (HMR); (b) full manure recycling scenario (FMR); (c) the scenario combining half manure recycling with manure-N fertilizer equivalency improved to 50% (HMR+MFE50); (d) the scenario combining full

manure recycling with manure-N fertilizer equivalency improved to 50% (FMR+MFE50). Map lines delineate study areas and do not necessarily depict accepted national boundaries.

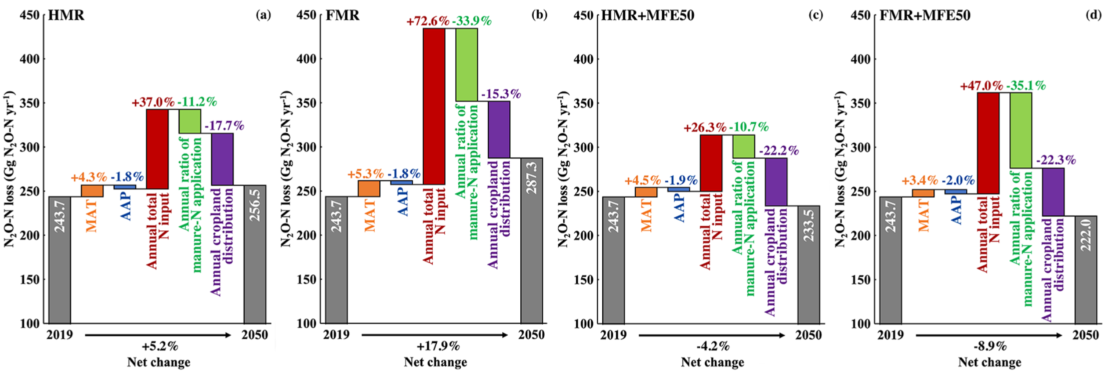

Figure S3. Contributions of drivers to changes in China's N_2O-N loss during 2019–2050 under each enhanced scenario. (a) half manure recycling scenario (HMR); (b) full manure recycling scenario (FMR); (c) the scenario combining half manure recycling with manure-N fertilizer equivalency improved to 50% (HMR+MFE50); (d) the scenario combining full manure recycling with manure-N fertilizer equivalency improved to 50% (FMR+MFE50). The length of each bar reflects the contribution of each driver to changes in N_2O-N losses during the corresponding period. Annual mean air temperature (MAT); annual accumulated precipitation (AAP); annual total N (the sum of chemical-N and manure-N) input (annual total N input); and the annual ratio of manure-N to total N (the sum of chemical-N and manure-N) application (annual ratio of manure-N application).

Supporting Table

Table S1. Performance comparison of different modeling approaches.

Model approach	R^2	RMSE (kg N_2O-N ha^{-1})	MAE (kg N_2O-N ha^{-1})
Generalized linear model	0.32	29.4	16.6
Random forest model	0.65	23.2	11.0
Boosted regression tree model	0.77	3.2	1.3

Regression coefficients of determination (R^2); root mean square error (RMSE); mean absolute error (MAE).

Table S2. N_2O-N emission factors (EFs) and key characteristics of six agricultural regions in China.

Region	EF (%) Median (standard deviation) *n*=simple size			MAT (°C)	AAP (mm)	TN (g/kg)	TP (g/kg)	AP (mg/kg)	pH	SOM (g/kg)	BD (g/cm^3)
	N source	Paddy	Upland								
Northeast	Chemical-N	0.2 (0.4) *n*=21	0.3 (0.6) *n*=72	5.1	691.7	2.0	0.8	6.9	6.7	38.0	1.2
	Mixed-N	NA	0.6 (0.6) *n*=32								
	Manure-N	NA	0.1 (0.0) *n*=3								
Northcentral	Chemical-N	0.5 (0.1) *n*=8	0.4 (1.4) *n*=549	9.8	506.0	0.9	0.6	4.6	8.0	13.9	1.3
	Mixed-N	NA	0.4 (0.7) *n*=241								
	Manure-N	NA	0.2 (0.5) *n*=89								
Northwest	Chemical-N	0.4 (0.1) *n*=10	0.4 (0.3) *n*=65	8.5	276.7	0.7	0.6	5.2	8.3	10.9	1.3
	Mixed-N	NA	0.5 (0.4) *n*=27								
	Manure-N	NA	0.3 (0.7) *n*=6								
Middle and lower of Yangtze river	Chemical-N	0.2 (0.6) *n*=248	0.8 (1.4) *n*=267	17.2	1219.9	1.5	0.5	5.0	5.6	27.7	1.2
	Mixed-N	0.1 (0.6) *n*=71	0.6 (1.0) *n*=96								
	Manure-N	0.1 (0.6) *n*=11	0.4 (1.0) *n*=44								
Southeast	Chemical-N	0.5 (0.4) *n*=20	2.2 (3.6) *n*=43	21.4	1682.3	1.2	0.4	4.6	5.2	28.2	1.2
	Mixed-N	NA	1.2 (7.4) *n*=12								
	Manure-N	0.05 (0.1) *n*=14	0.6 (0.6) *n*=4								

Region	EF (%) Median (standard deviation) *n*=simple size			MAT (°C)	AAP (mm)	TN (g/kg)	TP (g/kg)	AP (mg/kg)	pH	SOM (g/kg)	BD (g/cm^3)
	N source	Paddy	Upland								
Southwest	Chemical-N	0.3 (0.7) *n*=40	0.7 (1.4) *n*=144	15.8	980.6	1.7	0.5	4.8	6.0	32.9	1.2
	Mixed-N	NA	0.8 (1.4) *n*=27								
	Manure-N	NA	0.6 (0.4) *n*=10								
Overall	Overall	0.2 (0.6) *n*=445	0.5 (1.5) *n*=1735	13.3	730.6	1.4	0.6	5.0	6.7	26.3	1.2

EFs were derived from our field database. Climate and soil parameters are regional medians extracted from National Tibetan Plateau/Third Pole Environment Data Center and Harmonized World Soil Database (HWSD v2.0, 2023). NA indicates no observations available. Emission factor (EF); annual mean air temperature (MAT); annual accumulated precipitation (AAP); soil total nitrogen (TN); soil total phosphorus (TP); soil available phosphorus (AP); soil pH value (pH); soil organic matter (SOM); soil bulk density (BD); chemical-N application only (chemical-N); mixed chemical-N and manure-N application (mixed-N); manure-N application only (manure-N).

Table S3. Comparison of N_2O-N emission factors (EFs) across published empirical models

Number	Emission factor	Reference
1	(i) Paddy 0.2%; Upland 0.5% (ii) Northeast (chemical-N: paddy 0.2%/upland 0.3%; mixed-N: upland 0.6%; manure-N: upland 0.1%); Northcentral (chemical-N: paddy 0.5%/upland 0.4%; mixed-N: upland 0.4%; manure-N: upland 0.2%); Northwest (chemical-N: paddy 0.4%/upland 0.4%; mixed-N: upland 0.5%; manure-N: upland 0.3%); Middle and lower of Yangtze river (chemical-N: paddy 0.2%/upland 0.8%; mixed-N: paddy 0.1%/upland 0.6%; manure-N: paddy 0.1%/upland 0.4%); Southeast (chemical-N: paddy 0.5%/upland 2.2%; mixed-N: upland 1.2%; manure-N: paddy 0.05%/upland 0.6%); Southwest (chemical-N: paddy 0.3%/upland 0.7%; mixed-N: upland 0.8%; manure-N: upland 0.6%)	This study (derived from our field database)
2	Paddy 0.3%; Upland 1.0%	FAO
3	Paddy 0.4%; Upland 1.1%	NUFER
4	Northeast (paddy 1.6%; upland 1.5%); Northcentral (paddy 0.6%; upland 0.8%); Northwest (paddy 0.5%; upland 0.7%); Middle and lower of Yangtze river (paddy 1.1%; upland 1.6%); Southeast (paddy 0.6%; upland 0.9%); Southwest (paddy 0.6%; upland 0.9%)	Liang et al., 2024
5	Northeast (chemical-N: paddy 0.4%/upland 0.4%; mixed-N: paddy -/upland 0.4%; manure-N: paddy -/upland 0.3%); Northcentral (chemical-N: paddy -/upland 0.3%; mixed-N: paddy -/upland 0.3%; manure-N: paddy -/upland 0.3%); Northwest (chemical-N: paddy 0.4%/upland 0.6%; mixed-N: paddy -/upland 0.5%; manure-N: paddy -/upland 0.04%); Middle and lower of Yangtze river (chemical-N: paddy 0.3%/upland 0.7%; mixed-N: paddy 0.1%/upland 0.8%; manure-N: paddy 0.1%/upland 0.3%); Southeast and Southwest (chemical-N: paddy 0.7%/upland 0.5%; mixed-N: paddy 0.9%/upland 0.8%; manure-N: paddy -/upland 0.4%)	Yue et al., 2019
6	Northeast (chemical-N 0.6%; manure-N 1.0%); Northcentral (chemical-N 1.2%; manure-N 1.0%); Northwest (chemical-N 0.9%; manure-N 1.0%); Middle and lower of Yangtze river (chemical-N 1.0%; manure-N 1.0%); Southeast (chemical-N 1.0%; manure-N 1.0%); Southwest (chemical-N 0.8%; manure-N 1.0%)	He et al., 2018
7	Paddy 0.4%; Upland 1.1%	Gao et al., 2011

Chemical-N application only (chemical-N); mixed chemical-N and manure-N application (mixed-N); manure-N application only (manure-N).

Supporting Reference

Bai, Z., X. Fan, X. Jin, et al. 2022. “Relocate 10 Billion Livestock to Reduce Harmful Nitrogen Pollution Exposure for 90% of China's Population”. *Nature Food* 3: 152–160. https://doi.org/10.1038/s43016-021-00453-z.

Bai, Z., L. Ma, S. Jin, et al. 2016. “Nitrogen, Phosphorus, and Potassium Flows through the Manure Management Chain in China”. *Environmental Science & Technology* 50: 13409–13418. https://doi.org/10.1021/acs.est.6b03348.

Balkovič, J., M. van der Velde, R. Skalský, et al. 2014. “Global Wheat Production Potentials and Management Flexibility Under the Representative Concentration Pathways”. *Global and Planetary Change* 122: 107-121. https://doi.org/10.1016/j.gloplacha.2014.08.010.

Breiman, L. 2001. “Random Forests”. *Machine Learning* 45: 5-32. https://doi.org/10.1023/a:1010933404324.

FAOSTAT. 2024. *Food and Agriculture Data*. Food and Agriculture Organization. https://www.fao.org/faostat/.

Friedman, J. H. 2001. “Greedy Function Approximation: A Gradient Boosting Machine”. *Annals of Statistics* 29: 1189–1232. https://www.jstor.org/stable/2699986.

Friedman, J. H. 2002. “Stochastic Gradient Boosting”. *Computational Statistics & Data Analysis* 38: 367–378. https://doi.org/10.1016/S0167-9473(01)00065-2.

Herrero, M., P. K. Thornton, D. Mason-D'Croz, et al. 2021. “Articulating the Effect of Food Systems Innovation on the Sustainable Development Goals”. *The Lancet Planetary Health* 5: e50-e62. https://doi.org/10.1016/S2542-5196(20)30277-1.

Jensen, E. 2013. Treatment and Management. John Wiley & Sons Ltd.

Jin, X., Z. Bai, O. Oenema, et al. 2020. “Spatial Planning Needed to Drastically Reduce Nitrogen and Phosphorus Surpluses in China's Agriculture”. *Environmental Science & Technology* 54: 11894–11904. https://doi.org/10.1021/acs.est.0c00781.

Ma, L., W. Q. Ma, G. L. Velthof, et al. 2010. “Modeling Nutrient Flows in the Food Chain of China”. *Journal of Environmental Quality* 39: 1279-1289. https://doi.org/10.2134/jeq2009.0403.

Nelder, J. A., and R. W. Wedderburn. 1972. “Generalized Linear Models”. *Journal of the Royal Statistical Society Series A: Statistics in Society* 135: 370-384. https://doi.org/10.1016/S2542-5196(20)30277-1.

Takayama, T., H. Hashimoto, and N. D. Uri. 1984. “Spatial and Temporal Price and Allocation Modeling”. *Socio-Economic Planning Sciences* 18: 227-234. https://doi.org/10.1016/0038-0121(84)90002-8.

Tian, H., Z. Bian, H. Shi, et al. 2022. “History of Anthropogenic Nitrogen Inputs (HaNi) to the Terrestrial Biosphere: a 5 Arcmin Resolution Annual Dataset from 1860 to 2019”. *Earth System Science Data* 14: 4551–4568. https://doi.org/10.5194/essd-14-4551-2022.

Webb, J., P. Sørensen, G. Velthof, et al. 2013. “An Assessment of the Variation of Manure Nitrogen Efficiency throughout Europe and an Appraisal of Means to Increase Manure-N Efficiency”. *Advances in Agronomy* 119: 371–442. https://doi.org/10.1016/B978-0-12-407247-3.00007-X.

Williams, J., C. Jones, J. Kiniry, and D. A. Spanel. 1989. “The EPIC Crop Growth Model”. *Transactions of the ASAE* 32: 497-0511. https://doi.org/10.13031/2013.31032.

Zhao, H., J. Chang, P. Havlík, et al. 2021. “China’s Future Food Demand and Its Implications for Trade and Environment”. *Nature Sustainability* 4: 1042-1051. https://doi.org/10.1038/s41893-021-

00784-6.
